# Reliability, Objectivity, Validity and Reference Levels of the Austrian Balance Check (ABC)—A Novel Balance Field Test for Children, Adolescents and Young Adults to Assess Static Balance

**DOI:** 10.3390/sports13010005

**Published:** 2025-01-02

**Authors:** Gerald Jarnig, Reinhold Kerbl, Mireille N. M. van Poppel

**Affiliations:** 1 Institute of Human Movement Science, Sport and Health, University of Graz, 8010 Graz, Austria; 2 Department of Pediatrics and Adolescent Medicine, LKH Hochsteiermark, 8700 Leoben, Austria

**Keywords:** fitness, field test, balance, children, adolescents, reference value

## Abstract

Balance is a relevant indicator of physical fitness and correlates with intellectual abilities. Due to limited resources, however, balance tests are underrepresented in fitness checks. To develop an effective field test to assess static balance with minimal cost, little spatial requirement and short testing time, a cross-sectional pilot study was conducted in three different school types (primary school, secondary school, and high school) and the reliability, objectivity and validity of the novel Austrian Balance Check (ABC) were assessed, with the generation of age- and gender-specific reference values. Tests were carried out with participants from regular school classes (RSC) and elite sports school classes (ESC). A total of 1005 participants were included (age range: 6.2 to 19.8 years). Participants in RSC (ICC_2.1_ = 0.92, 95% CI 0.90 to 0.93) showed excellent values, and those in ESC (ICC_2.1_ = 0.89, 95% CI 0.85 to 0.93) had good to excellent values in the reliability test. Objectivity was excellent in both groups (RSC (ICC_2.1_ = 0.99, 95% CI 0.98 to 0.99); ESC (ICC_2.1_ = 0.98, 95% CI 0.96 to 0.99)). A gold standard comparison using an electronic force plate showed a strong negative correlation: participants with high overall ABC scores showed less movement on the force plate (parameters of the center of pressure: surface area (ρ = −0.61), mean velocity (ρ = −0.65) and path length (ρ = −0.65). The test duration of ABC was significantly (*p* < 0.001) shorter compared with an established balance test. ABC offers potential benefits by allowing field-based static balance testing in large groups with low cost, minimal time and spatial requirements as well as high reliability, objectivity and validity.

## 1. Introduction

The vestibular system—as the human organ of balance—sends enormous amounts of information to the brain [1,2]. All sensory perceptions are processed involving the vestibular information received [3]. It has been shown that good balancing ability correlates with increased cognitive abilities [4]. For example, there are high correlations between children’s poor reading or spelling skills and low motor balance performance [5,6]. Regardless of age or gender, better motor balance also correlates with higher fitness parameters such as strength, speed, coordination, agility or endurance [7,8,9,10]. Additionally, good balance increases the efficiency and effectiveness of movements and activities and reduces the risk of injury [11]. Adding warm-up programs that include balance exercises and other fitness parameters such as flexibility or strength to everyday training routines reduces the potential risk of injuries by at least 35% [12].

The World Health Organization called increasingly for counteracting the trend of declining physical fitness levels in children and adolescents that have been observed over the last few decades [13]. As a consequence, the documentation and evaluation of physical fitness was intensified and fitness development was observed over longer periods of time [14,15,16]. However, the assessment of balance is often not part of international test batteries [14,17,18,19]. In reviews of field tests assessing physical fitness in children and adolescents, balance was either not included or reported. Only 3 of 84 (=3.6%) selected papers consider this parameter [20].

This is surprising, as periodic balance monitoring is important for appropriate planning of training [21]. However, valid balance tests are either very cost-intensive and/or time-consuming [22,23,24]; it is often not practicable to carry out tests with large groups due to time limitations. Digital measurement of balance using portable force plates is an alternative option but is cost-intensive and therefore impractical in the field [25,26]. Therefore, alternative testing options are needed [27].

The aim of this work is to determine the test quality criteria of a newly designed innovative field test for the assessment of static balance and to generate age- and gender-specific reference values based on a large study population.

## 2. Materials and Methods

Prior to this study, the reliability and competitiveness of the newly designed balance field test were evaluated by testing a small study population [19]. Based on its findings, a cross-sectional pilot study was carried out at a school campus in Klagenfurt City, Austria. The study was approved by the Research Ethics Committee of the University of Graz, Styria, Austria (GZ. 39/68/63 ex 2021/22).

### 2.1. Selection of School Campus and Study Participants

At the school campus in Klagenfurt City, different types of schools (primary school, secondary school and high-school) are located in one building complex. All schools administrations agreed to take part in the cross-sectional study. In primary school, all children attend a general school branch; in secondary school and high school, a general (regular school classes (RSC)) and a sports-performance-oriented branch (elite sports classes (ESC)) are conducted in parallel. The following inclusion criteria were defined: the children had to attend one of the three schools on the campus, have a confirmed school-ready ability [28], and be able to complete all balance-specific tests without restrictions.

**Figure 1:**
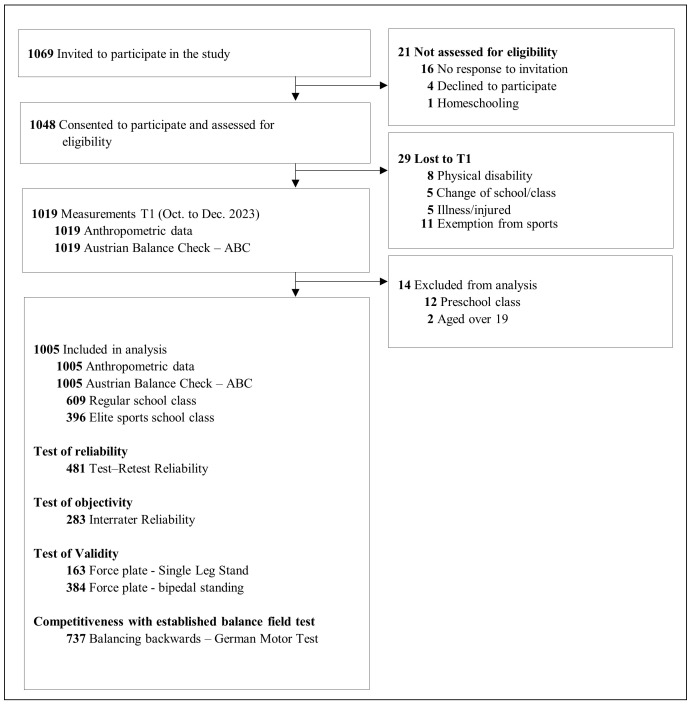
Flow diagram.

A total of 1069 children, adolescents and young adults were invited to take part in the study. The legal guardians of children aged 14 years and younger were informed in writing about the study content and asked to authorize their children’s participation. A total of 1048 (98.0%) potential study participants or their legal guardians agreed to participate in the study and provided information regarding age, gender and school type (Figure 1).

### 2.2. Procedure

The measurement of anthropometric data and balance was carried out by trained members of the research team and took place in the schools during physical education lessons. All tests were performed without shoes on a non-slippery surface, except the backward balancing test (wearing sports shoes), and the participants wore standard sportswear.

#### 2.2.1. Anthropometry

Height (cm) was measured to the nearest 0.1 cm using a SECA 213 stadiometer and weight (kg) was measured to the nearest 0.1 kg using a PPW4202/01 body scale (Bosch, Munich, Germany). The BMI was calculated by dividing the body weight in kg by the height in meters squared.

#### 2.2.2. Austrian Balance Check (ABC)

**Figure 2:**
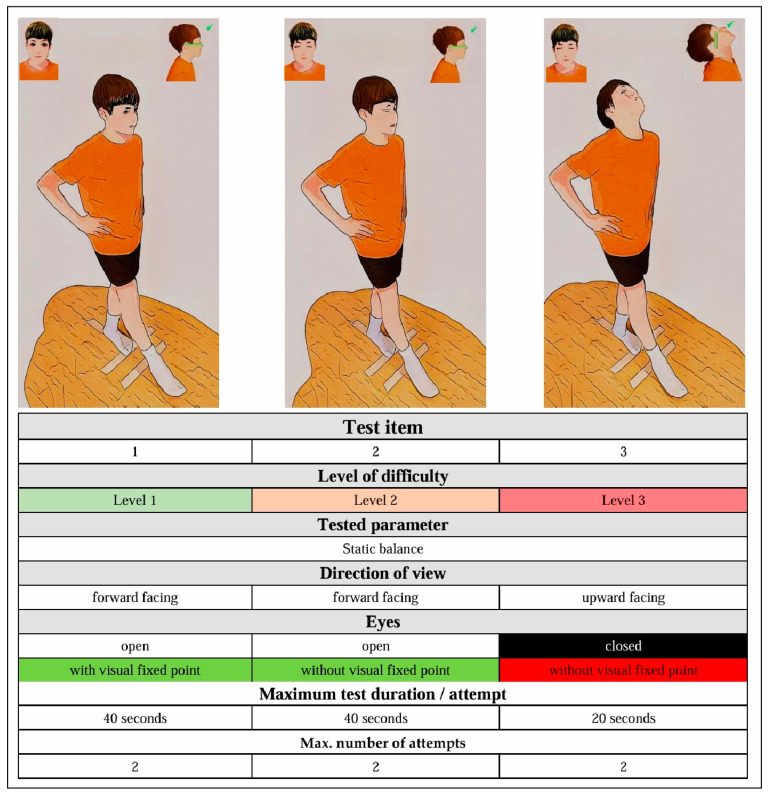
Correct body position, execution, and key points of the Austrian Balance Check.

The Austrian Balance Check (ABC) is a three-stage field test, with increasing levels of difficulty (Figure 2) to assess static balance.

Participants stand with their preferred leg on a marker, both hands on the hips and the other leg moved forward and up.

To move on to the next level of difficulty, participants must first successfully complete the previous level. The maximum time for a level must be completed without making any mistakes (Level 1&2 = 40 s; Level 3 = 20 s). Each participant has a maximum of two attempts per level; if the first attempt at level 1 or 2 is successful, no second attempt will take place at this level and the level is marked as successfully completed on the assessment sheet. Participants who reach level 3 must always complete two attempts at this level. Participants who are unable to maintain the correct body position for the full 40 s during one of their two attempts either in level 1 or 2 must discontinue the ABC at that point. An attempt ends when the maximum time to complete a level has been reached, one hand loses hip contact, the standing leg leaves the mark on the floor, the free leg is moved backward, the eyes are opened in levels 2 and 3, or the chin moves forward and downward in level 3. Detailed information about the correct body position during the test procedure at the different levels is given in Figure 2 and can be found in the ABC Test Manual in the additional material.

For each level (1 or 2) successfully completed, participants receive 7 points. An additional point per level (only levels 1 and 2) can be collected if the participant is able to successfully complete the level by the first attempt. If a level is not successfully completed, the participant receives one point for every 10 s of maintaining the correct body position. The points resulting from both incomplete attempts are added to the overall assessment. This also applies to level 3, where participants must complete both attempts, regardless of whether the first attempt was successful or not. A maximum of 20 points can be achieved in total (practical examples and more detailed information about carrying out the ABC can be found in the supplements—Additional Methods 1).

#### 2.2.3. Gold Standard Comparison—Single Leg Stand Test Using the KNIVENT Force Plate (FP SLS)

A validated KINVENT force plate system (SÜSS Medical Technologies, Tumeltsham, Austria) [29] with an electronic pressure transducer consisting of two portable force plates (dimensions per plate are 30 × 346 × 191 mm, weight 2.0 kg, maximum force per plate 600 kg) was used. Audio and visual biofeedback are sent to a smartphone in real time via the KINVENT application (Kinvent Biomechanics, Starter license, version 2.6.1, Montpellier, France). The acquisition frequency is 1000 Hz and the Kinvent sensors use the 2.4 GHz band (Bluetooth Low Energy 5.1) as the wireless transmission frequency with a maximum radiated power of 10 mW.

A self-adapted version of the single-leg stand test was used to measure static balance with eyes open and eyes closed. In the starting position, children stand with their legs in the middle of the force plate while their hands are on their hips. After an acoustic signal, the children raise their playing leg up and forward and hold this position for 5 s. Three repetitions are completed per leg, with a 7 s break between. The test is performed with eyes open and then repeated with eyes closed. The raw data of the center of pressure (COP) (mean COP surface area in mm^2^ (COP Su), mean COP velocity in mm/s (COP Mv), and COP distance in mm (COP Pl)) of the measurements with open and closed eyes of the preferred leg (standing leg selected at ABC) were exported from the Knivent software application (Kinvent Biomechanics, Starter license, version 2.6.1, Montpellier, France) for analysis and included in the overall assessment.

#### 2.2.4. Competitiveness—Backward Balance Test According to Bös (BB GMT)

The test was carried out in accordance with the Bös test manual [30]. The participants walk backward over three different beams (6, 4.5 and 3 cm wide, length = 3.00 m). Two attempts must be completed on each beam. The number of steps taken on the beam without touching the floor is recorded. The maximum number of steps to be achieved per round is set at 8. If a participant can successfully complete a beam with fewer steps, 8 steps will be documented as the number for this attempt. The sum of the number of steps from all 6 attempts was included in the overall assessment. Additionally, the total time required to test a group was documented and then divided by the number of participants in that group, and the average time required to test a child was included in the overall assessment.

### 2.3. Grouping, Standardization and Classification

Participants were categorized into 7 age groups (≤7.9 years, 8.0 to 9.9 years, 10.0 to 11.9 years, 12.0 to 13.9 years, 14.0 to 15.9 years, 16.0 to 17.9 years and 18.0 to 19.9). These two-year steps were defined to ensure larger group sizes for calculating the reference values while taking into account the school organization and the limited number of participants. Gender-specific means and standard deviations were calculated for all variables.

National reference values were used for BMI standardization and weight classification [31]. More detailed information about the weight classification is given in the Supplementary Materials (Additional Methods 2 in the Supplementary Materials).

Using the traditional z-score standardization [32], z-values were calculated for all achievable total points (x = 0 to 20) in the different age groups and for both genders.

$${\text{z-score}}_{x}=\frac{X-M_{agegroup\&sex}}{SD_{agegroup\&sex}}$$

Variables:

x = Total points from ABC,

$M_{agegroup\&sex}$ = Age- and gender-specific mean value,

$SD_{agegroup\&sex}$ = Age- and gender-specific standard deviation.

Z-scores of the achievable total points were then converted into a nine-point score (STA9) with a mean of 5 and a standard deviation of 2 [33].

$${\text{STA9}}_{\mathrm{Raw}\mathrm{value}x}=5+(2*{\text{z-score}}_{x})$$

The STA9 values were categorized into a nine-point rating (from poor (category 1) to excellent (category 2)) (Table S1).

In order to assess the test–retest reliability of the ABC, 342 participants were tested twice by the same test administrator (interrater reliability), with a one- to two-week interval between testing sessions.

Objectivity was assessed using interrater reliability, with two test administrators simultaneously and independently observing, documenting and evaluating 210 participants’ performance in ABC.

Laboratory-based assessment using center of pressure (COP) measurements recorded from a force platform (FP) represents the gold standard for assessing human balance [25,34]. To assess validity, participants performed the ABC, and on the same day, COP data (surface area, mean velocity, path length) were measured using a force plate.

In order to assess competitiveness on a large population, execution times and performance of an established balance field test were compared with data from the ABC.

To test reliability and objectivity, those participants and administrators who were available again within two weeks were selected.

A random generator was used to select one class of the regular school branch per school grade for testing validity. More detailed information about the selection of participants is given in the Supplementary Materials (Additional Methods 3 in the Supplementary Materials).

The competitiveness check was carried out within a time window of 3 weeks by all participants who were organizationally and health-wise able to take part in the test.

### 2.4. Statistical Analysis

Continuous variables are expressed as mean (M) and standard deviation (SD), with categorical variables as absolute values (n) and percentages (%) for descriptive statistics.

Independent *t*-tests were performed to identify differences between groups (RSC vs ESC; boys vs girls). A Mann–Whitney U-test was used to determine the differences in BMI categories and ABC performance classifications between the groups.

Interrater reliability was calculated for analyzing reliability and objectivity using a two-sided mixed intraclass correlation coefficient (ICC) based on individual measures and absolute agreement for the raw scores of the ABC [35]. To define reliability, the 95% CIs of the ICCs were interpreted as follows: 95% CI values below 0.5 were considered poor reliability, values between 0.5 and 0.75 as moderate reliability, values between 0.75 and 0.9 as good reliability, and values above 0.90 as excellent reliability.

To determine the validity, the Spearman correlation coefficient (ρ) between ABC performance and results measured with force plates was calculated. Correlations were classified according to Cohen [36], with a weak correlation as ρ ≥ 0.1, a medium correlation as ρ ≥ 0.3, and a strong correlation as ρ ≥ 0.5.

Paired *t*-tests were performed to compare the duration of the balance tests. In addition, the data were visually checked for normal distributions. No imputation of the data was performed. All statistical analyses were performed in SPSS 29.0 (IBM SPSS Statistics 29, IBM, New York, NY, USA) with a significance level of *p* < 0.05.

## 3. Results

Between September and December 2023, a total of 1019 participants completed anthropometric and balance measurements. Children who were assigned to a preschool class (n = 12) and participants older than 19 years (n = 2) were excluded from the analysis. Finally, the data from 1005 participants were used for analysis (Figure 1).

Among the total of 1005 participants, 609 participants (female = 53.0%) attended RSC and 396 participants (female = 22.2%) attended ESC (Table S2).

Significant differences (*p* < 0.001 [height, EQUI _BMIAUT_, raw scores ABC]; *p* = 0.002 [age]) between the participants of the RSC and ESC were detected (Tables S3 and S4); therefore, the data of the two different school branches were analyzed separately in all calculations.

The participants of the ESC showed significantly (*p* < 0.001) better performance in ABC (Table S3) than those of the RSC; therefore only data from students of the RSC were used to classify the results of the ABC, as a sports performance test is mandatory for entry into ESC and data from these participants would strongly bias results.

Raw data from ABC showed a continuous increase in mean scores from the youngest to the oldest age group. Girls performed better in all age groups, but a significant gender difference (*p* = 0.036) was only found in the youngest age group (Figure S1, Table S5). The gender- and age-specific reference values calculated from the traditional z-score standardization and resulting STA9 classification are presented in Table 1 (Table 1 and Table S6).

Reliability was excellent for all participants in the RSC (ICC = 0.92 (95% CI, 0.90–0.93)) and good to excellent in both boys (95% CI, 0.89–0.94) and girls (95% CI, 0.89–0.94). In the ESC, overall reliability was good to excellent (ICC = 0.89 (95% CI, 0.85–0.93)); the same reliability was also observed in the subgroups of boys and girls (Table 2). Objectivity was excellent in both the RSC (ICC = 0.99 (95% CI, 0.98–0.99)) and the ESC (ICC = 0.98 (95% CI, 0.96–0.99)) overall and for both genders (Table 2).

Strong negative correlations (COP Su = −0.61; COP Mv = −0.65; COP Pl = −0.65) were observed when testing the validity of the gold standard comparison between performance at ABC and COP parameters measured using the force plate. This means that participants with higher scores on ABC demonstrated less movement on the electronic force plate. Comparable results were also found in the subgroups of boys and girls (Table 3).

Additional detailed age- and gender-specific information are presented in the supplements (Tables S7–S11).

Assessment of competitiveness with an established field test for generally assessing balance (backward balancing according to Bös) showed a strong correlation of the raw data in the RSC across all participants; a moderate correlation was observed in the ESC. The expenditure of ABC testing was significantly (*p* < 0.001) shorter (RSC = 1.28 min per participant; ESC = 1.29 min per participant) than the backward balancing test (Tables S2, S12 and S13). The visual check between the classification of both balance field tests for normal distribution showed a more satisfying result for ABC from the subjective point of view of the authors. Especially in the ESC, a clear ceiling effect was seen in the better performance categories in the BB (Figure S2).

## 4. Discussion

The results of the ABC balance check are in line with international reports and reference values showing an increase in balance performance with age and better performance in girls [24,37,38].

The ABC was shown to be reliable and valid and therefore suitable for all kinds of settings. Excellent inter-rater and intra-rater reliability underline the test’s outstanding practicality. These values are comparable to established fitness tests used in countless studies worldwide to assess different fitness parameters, such as the 6-min walk, the standing long jump or the medicine ball throw [18]. Furthermore, they confirm that the ABC generates reliable results independently, regardless of the test administrator or testing time. This high degree of reproducibility and objectivity is a strong argument for using the test also in research and establishes it as a very useful addition to existing fitness tools.

Reference values for school-age children up to the age of 19 developed in this study provide excellent opportunities for practical use in the field, which can be extended by further studies. It is well documented that older people lose balance performance over their lifetime [39,40]. The ABC could also be a valuable tool for identifying such a decline and contributing to the evaluation of any counteraction efforts. Of course, age-specific reference values must first be established for this purpose by future studies. Another potential of the ABC is seen in the testing of physically or intellectually [41] disabled persons, who also frequently demonstrate reduced balance performance.

Results from this and the concept study in 2022 [19] indicate that ABC in comparison with other balance field tests shows better fitting to normal distribution. No ceiling effect was observed and the time needed for test performance is significantly shorter than for established tests. Tests without a ceiling effect offer several advantages, in particular, due to their ability to differentiate and the associated improved significance compared to tests with expected ceiling effects. This is directly linked to the increased suitability for testing heterogeneous groups and collecting more precise data in long-term observations.

The gold-standard comparison between the force plate and the ABC shows a strong correlation (COP Su = −0.61; COP Mv = −0.65; COP Pl = −0.65), although presenting different results. The correlation values are comparable to other study results, which report correlations between movement-specific field tests with laboratory tests [42,43,44].

The importance of balance in relation to cognitive and motor abilities has been shown [4,5,7,8,9,10,22]; however, only a few international test batteries have included this parameter thus far. The novel ABC could contribute to more inclusion of the parameter “balance” in test batteries.

An important reason why balance tests are often not included in test batteries is the fact that ceiling effects often exist in the higher performance categories. This significantly limits the usefulness of balance tests in longitudinal studies. When comparing the results of the ABC and BB in the ESC, we can see this ceiling effect in the BB but not the ABC. The argument against the use of balance tests in test batteries and long-term studies is invalidated by the development of the ABC. ABC is a suitable monitoring tool for evaluating balance without a high ceiling effect.

The ABC is an ideal tool to efficiently test balance in large groups, as it can be carried out with minimal cost, minimal space requirements and in a short time. The existing reference values and the simple assessment method enable a reliable interpretation of the results, even by persons without specialized medical or sports science knowledge. This is another benefit that further highlights the practicality and usefulness of this test.

### Strengths and Limitations

There are several strengths. The test quality criteria were checked with a large number of participants, an electrical force plate was used to create a gold standard comparison, the test was easy to use (test duration, no ceiling effect), and age- and gender-specific reference values were developed. In addition, a more detailed test manual is presented in the Supplementary Materials.

Limitations are the small numbers of participants in each age group, age groups of two years instead of one year age span, and the fact that all data were collected at one site. Larger studies including more regions are needed, in order to be able to calculate age-specific reference values with a possibly higher degree of reliability.

## 5. Conclusions

ABC is a reliable, valid and time-saving balance check. No ceiling effects were observed. Therefore, it has the potential to close a gap in existing science by allowing field-based testing of static balance for large groups at low costs and with minimal spatial requirements. This could give way to more and better inclusion of balance testing in different test batteries and settings.

## Figures and Tables

**Table 1:** Age- and gender-specific reference values for school-age participants in the Austrian Balance Check.

Variable			Gender	Age Group	Total Points Achieved (0 to 20) in the Austrian Balance Check																				
					0	1	2	3	4	5	6	7	8	9	10	11	12	13	14	15	16	17	18	19	20
Allocation of the total points to the performance categories			Male	≤7	1	2	3	3	4	5	5	6	6	7	8	8	9	9	9	9	9	9	9	9	9
				8 to 9	1	2	2	3	3	4	4	5	5	6	6	6	7	7	8	8	9	9	9	9	9
				10 to 11	1	1	2	2	2	3	3	4	4	5	5	6	6	6	7	7	8	8	9	9	9
				12 to 13	1	1	1	1	1	2	2	3	3	4	4	5	5	6	7	7	8	8	9	9	9
				14 to 15	1	1	1	1	1	1	2	2	3	4	4	5	5	6	6	7	7	8	9	9	9
				16 to 17	1	1	1	1	1	1	1	2	2	3	3	4	5	5	6	7	7	8	9	9	9
				18 to 19	1	1	1	1	1	1	1	1	2	2	3	4	4	5	6	6	7	8	8	9	9
			Female	≤7	1	2	2	3	3	4	4	5	5	6	6	7	7	8	8	9	9	9	9	9	9
				8 to 9	1	1	2	2	3	3	4	4	5	5	6	6	7	7	8	8	9	9	9	9	9
				10 to 11	1	1	1	1	1	1	2	3	3	4	5	5	6	7	8	8	9	9	9	9	9
				12 to 13	1	1	1	1	1	1	2	2	3	3	4	5	5	6	6	7	8	8	9	9	9
				14 to 15	1	1	1	1	1	1	1	2	3	3	4	4	5	6	6	7	8	8	9	9	9
				16 to 17	1	1	1	1	1	1	1	2	2	3	3	4	5	5	6	6	7	7	8	8	9
				18 to 19	1	1	1	1	1	1	1	1	2	2	3	4	4	5	5	6	6	7	8	8	9
Categories of classification																									
Low balance performance							Average balance performance									High balance performance									
1		Poor balance					4		Below-average balance							7		Good balance							
2		Very weak balance					5		Average balance							8		Very good balance							
3		Weak balance					6		Above-average balance							9		Excellent balance							

**Table 2:** Reliability and objectivity of ABC performances for all participants and separately for boys and girls.

Test Quality Criteria	Group	Regular School Class					Elite Sports School Class				
		N	ICC (2.1)	95% CI		Agreement	N	ICC (2.1)	95% CI		Agreement
				Lower	Upper				Lower	Upper	
Test of Reliability	All	342	0.919	0.900	0.934	Excellent	139	0.894	0.850	0.925	Good to Excellent
	Boys	161	0.920	0.892	0.941	Good to Excellent	104	0.891	0.836	0.927	Good to Excellent
	Girls	181	0.913	0.885	0.935	Good to Excellent	35	0.905	0.821	0.951	Good to Excellent
Test of Objectivity	All	210	0.985	0.980	0.988	Excellent	73	0.975	0.961	0.985	Excellent
	Boys	110	0.979	0.970	0.986	Excellent	52	0.969	0.947	0.982	Excellent
	Girls	100	0.992	0.988	0.994	Excellent	21	0.995	0.989	0.998	Excellent

Explanation: To determine reliability, test [ABC T1]—ReTest [ABC T2]) and objectivity (Test rater 1 [ABC R1 T1]—Test rater 2 [ABC R2 T2]), the 95% CIs of the ICCs were interpreted as follows: 95% CI values below 0.5 were considered to indicate poor reliability, values between 0.5 and 0.75 were considered to indicate moderate reliability, values between 0.75 and 0.9 were considered to indicate good reliability and values above 0.90 were considered to indicate excellent reliability. ICC = intraclass correlation, CI = confidence interval, ABC = Austrian Balance Check, T1 = Baseline measurements made in autumn 2023, T2 = Measurement taken within 2 weeks of the baseline measurement, R1 = Rater 1, R2 = Rater 2.

**Table 3:** Checking for validity using Spearman’s correlation coefficient.

	Variable			Regular School Class			
				ABC rs	FP SLS. rs		
					COP Su, mm^2^	COP Mv. mm/s	COP Pl. mm
School-age children & adolescents up to 19 years	All	ABC rs		--			
		FP SLS. rs	Surface. mm^2^	−0.612 **	--		
			COP Mv. mm/s	−0.653 **	0.678 **	--	
			COP Pl. mm	−0.651 **	0.679 **	0.999 **	--
	Boys	ABC rs		--			
		FP SLS. rs	Surface. mm^2^	−0.589 **	--		
			COP Mv. mm/s	−0.585 **	0.710 **	--	
			COP Pl. mm	−0.590 **	0.714 **	0.996 **	--
	Girls	ABC rs		--			
		FP SLS. rs	Surface. mm^2^	−0.570 **	--		
			COP Mv. mm/s	−0.620 **	0.594 **	--	
			COP Pl. mm	−0.615 **	0.593 **	0.999 **	--

** = Correlation is significant at the 0.01 level (2-tailed); ABC = Austrian Balance Check, T3 = Measuring timing of ABC on the same day as measuring of balance using an electronic force plate; rs = raw scores, FB = force plate, SLS = single leg stand, COP = center of pressure, Su = surface, Mv = Medium velocity, Pl = Path length, mm^2^ = square millimeter, mm/s = millimeters per second.

## Data Availability

The data presented in this study are available on request from the corresponding author. The data are not publicly available due to privacy/ethical restrictions.

## Supplementary Materials

The following supporting information can be downloaded at https://www.mdpi.com/article/10.3390/sports13010005/s1: Additional Methods 1: Test–Manual–Austrian Balance Check (ABC); Additional Methods 2: Weight classification; Additional Methods 3: Selection of participants for checking test quality criteria and competitiveness; Table S1. Classification of Austrian Balance Check; Table S2. Overall sample characteristics; Table S3. Differences between regular school classes and elite sports classes; Table S4. Mann–Whitney U-test used to assess differences in classification groups between regular school classes and elite sports classes; Table S5. Gender- and age-specific mean values of raw values of Austrian Balance Check; Table S6. Traditional z-scores and STA nine values; Table S7. Descriptive statistics of the results for checking objectivity; Table S8. Descriptive statistics of the results for checking reliability using the test–retest method; Table S9. Reliability and objectivity of ABC performance in different age groups and separately for boys and girls; Table S10. Descriptive statistics of the results for checking validity using the KINVENT force plate; Table S11. Age-specific data from the validity check using Spearman's correlation coefficient; Table S12. Age- and gender-specific data from the competitiveness review using Spearman's correlation coefficient; Table S13. Comparison of testing times between ABC and BB GMT by using paired *t*-test; Figure S1. Gender- and age-specific mean values of raw values of Austrian Balance Check; Figure S2. A graphical illustration of the normal distribution using histograms for the classification of the Austrian Balance Check and balancing backwards (German Motor Test).

## References

[1] Day B.L., Fitzpatrick R.C. (2005). The vestibular system. Curr. Biol..

[2] Fitzpatrick R.C., Day B.L. (2004). Probing the human vestibular system with galvanic stimulation. J. Appl. Physiol..

[3] Ayres A.J. (2016). Bausteine der Kindlichen Entwicklung: Sensorische Integration Verstehen und Anwenden—Das Original in Moderner Neuauflage.

[4] Abu–Shihab E.N., Abu Mohammad M.F., Khazaleh W.M., Bataineh A.S. (2017). Academic Achievement and Anthropometric Measurements and their Correlation with the Ability of Motor Balance and Concentration for 12 Year-Old Children. J. Educ. Psychol. Sci..

[5] Duff D.M., Hendricks A.E., Fitton L., Adlof S.M. (2022). Reading and Math Achievement in Children with Dyslexia, Developmental Language Disorder, or Typical Development: Achievement Gaps Persist From Second Through Fourth Grades. J. Learn. Disabil..

[6] Nicolson R.I., Fawcett A.J., Dean P. (2001). Developmental dyslexia: The cerebellar deficit hypothesis. Trends Neurosci..

[7] Acar H., Eler N. (2019). The Effect of Balance Exercises on Speed and Agility in Physical Education Lessons. Univers. J. Educ. Res..

[8] Gebel A., Prieske O., Behm D.G., Granacher U. (2020). Effects of Balance Training on Physical Fitness in Youth and Young Athletes: A Narrative Review. Strength Cond. J..

[9] Hrysomallis C. (2011). Balance ability and athletic performance. Sports Med..

[10] Nakano M.M., Otonari T.S., Takara K.S., Carmo C.M., Tanaka C. (2014). Physical performance, balance, mobility, and muscle strength decline at different rates in elderly people. J. Phys. Ther. Sci..

[11] Conner B.C., Petersen D.A., Pigman J., Tracy J.B., Johnson C.L., Manal K., Miller F., Modlesky C.M., Crenshaw J.R. (2019). The cross-sectional relationships between age, standing static balance, and standing dynamic balance reactions in typically developing children. Gait Posture.

[12] Emery C.A., Pasanen K. (2019). Current trends in sport injury prevention. Best Pract. Res. Clin. Rheumatol..

[13] Bull F.C., Al-Ansari S.S., Biddle S., Borodulin K., Buman M.P., Cardon G., Carty C., Chaput J.-P., Chastin S., Chou R. (2020). World Health Organization 2020 guidelines on physical activity and sedentary behaviour. Br. J. Sports Med..

[14] Bianco A., Jemni M., Thomas E., Patti A., Paoli A., Ramos Roque J., Palma A., Mammina C., Tabacchi G. (2015). A systematic review to determine reliability and usefulness of the field-based test batteries for the assessment of physical fitness in adolescents-The ASSO Project. Int. J. Occup. Med. Environ. Health.

[15] Cvejić D., Pejović T., Ostojić S. (2013). Assessment of physical fitness in children and adolescents. Facta Univ. Ser. Phys. Educ. Sport.

[16] Fühner T., Kliegl R., Arntz F., Kriemler S., Granacher U. (2021). An Update on Secular Trends in Physical Fitness of Children and Adolescents from 1972 to 2015: A Systematic Review. Sports Med..

[17] Freedson P.S., Cureton K.J., Heath G.W. (2000). Status of Field-Based Fitness Testing in Children and Youth. Prev. Med..

[18] Bös K. (2017). Handbuch Motorische Tests: Sportmotorische Tests, Motorische Funktionstests, Fragebögen zur Körperlich-Sportlichen Aktivität und Sportpsychologische Diagnoseverfahren.

[19] Jarnig G., Kerbl R., vanPoppel M. (2024). Reliability and Competitiveness of a Novel Balance Field Test: A Cross-Sectional Pilot Study. medRxiv.

[20] Tabacchi G., Lopez Sanchez G.F., Nese Sahin F., Kizilyalli M., Genchi R., Basile M., Kirkar M., Silva C., Loureiro N., Teixeira E. (2019). Field-Based Tests for the Assessment of Physical Fitness in Children and Adolescents Practicing Sport: A Systematic Review within the ESA Program. Sustainability.

[21] Ricotti L. (2011). Static and dynamic balance in young athletes. J. Hum. Sport Exerc..

[22] Panjan A., Sarabon N. (2010). Review of Methods for the Evaluation of Human Body Balance. Sport Sci. Rev..

[23] Gilberto C., Di Alain D., Julia M., Adriana G.L.D.S., Vania Cristina D.R.M., José Elias T., Guillaume T. (2018). How to evaluate the postural balance in a more efficient and less expensive way?. Procedia CIRP.

[24] Condon C., Cremin K. (2014). Static balance norms in children. Physiother. Res. Int..

[25] Clark R.A., Bryant A.L., Pua Y., McCrory P., Bennell K., Hunt M. (2010). Validity and reliability of the Nintendo Wii Balance Board for assessment of standing balance. Gait Posture.

[26] Duarte M., Freitas S.M. (2010). Revision of posturography based on force plate for balance evaluation. Braz. J. Phys. Ther..

[27] Heidt C., Vrankovic M., Mendoza A., Hollander K., Dreher T., Rueger M. (2021). Simplified digital balance assessment in typically developing school children. Gait Posture.

[28] Niklas F., Cohrssen C., Vidmar M., Segerer R., Schmiedeler S., Galpin R., Klemm V.V., Kandler S., Tayler C. (2018). Early childhood professionals’ perceptions of children’s school readiness characteristics in six countries. Int. J. Educ. Res..

[29] Meras Serrano H., Mottet D., Caillaud K. (2023). Validity and Reliability of Kinvent Plates for Assessing Single Leg Static and Dynamic Balance in the Field. Sensors.

[30] Bös K. (2016). Deutscher Motorik-Test 6-18: (DMT 6-18): Manual und Internetbasierte Auswertungssoftware.

[31] Mayer M., Gleiss A., Häusler G., Borkenstein M., Kapelari K., Köstl G., Lassi M., Schemper M., Schmitt K., Blümel P. (2015). Weight and body mass index (BMI): Current data for Austrian boys and girls aged 4 to under 19 years. Ann. Hum. Biol..

[32] Schober P., Mascha E.J., Vetter T.R. (2021). Statistics From A (Agreement) to Z (z Score): A Guide to Interpreting Common Measures of Association, Agreement, Diagnostic Accuracy, Effect Size, Heterogeneity, and Reliability in Medical Research. Anesth. Analg..

[33] Dimitrov D.M. (2014). Statistical Methods for Validation of Assessment Scale Data in Counseling and Related Fields.

[34] Donath L., Roth R., Zahner L., Faude O. (2012). Testing single and double limb standing balance performance: Comparison of COP path length evaluation between two devices. Gait Posture.

[35] Koo T.K., Li M.Y. (2016). A Guideline of Selecting and Reporting Intraclass Correlation Coefficients for Reliability Research. J. Chiropr. Med..

[36] Cohen J. (1988). Statistical Power Analysis for the Behavioral Sciences.

[37] Franjoine M.R., Darr N., Held S.L., Kott K., Young B.L. (2010). The performance of children developing typically on the pediatric balance scale. Pediatr. Phys. Ther..

[38] Rival C., Ceyte H., Olivier I. (2005). Developmental changes of static standing balance in children. Neurosci. Lett..

[39] Osoba M.Y., Rao A.K., Agrawal S.K., Lalwani A.K. (2019). Balance and gait in the elderly: A contemporary review. Laryngoscope Investig. Otolaryngol..

[40] Nolan M., Nitz J., Choy N.L., Illing S. (2010). Age-related changes in musculoskeletal function, balance and mobility measures in men aged 30–80 years. Aging Male.

[41] Lahtinen U., Rintala P., Malin A. (2007). Physical performance of individuals with intellectual disability: A 30 year follow up. Adapt. Phys. Act. Q..

[42] Burke E.J. (1976). Validity of Selected Laboratory and Field Tests of Physical Working Capacity. Res. Q. Am. Alliance Health Phys. Educ. Recreat..

[43] Aandstad A. (2020). Association Between Performance in Muscle Fitness Field Tests and Skeletal Muscle Mass in Soldiers. Mil. Med..

[44] Bell M., Fotheringham I., Punekar Y.S., Riley J.H., Cockle S., Singh S.J. (2015). Systematic Review of the Association Between Laboratory- and Field-Based Exercise Tests and Lung Function in Patients with Chronic Obstructive Pulmonary Disease. Chronic Obstr. Pulm. Dis..

