# Supplementary material for: Reliability, Objectivity, Validity and Reference Levels of the Austrian Balance Check (ABC)—A Novel Balance Field Test for Children, Adolescents and Young Adults to Assess Static Balance
Source: Sports (Basel). 2025 Jan 2;13(1):5. doi: 10.3390/sports13010005 (PMC11769504; doi:10.3390/sports13010005)
Supplement: Supplementary file 1 [file sports-13-00005-s001.zip › sports-3350795-supplementary.pdf]

Reliability, Objectivity, Validity and Reference Levels of the Austrian Balance Check (ABC)—A Novel Balance Field Test for Children, Adolescents and Young Adults to Assess Static Balance

Supplementary Materials

Gerald Jarnig, Reinhold Kerbl, Mireille N. M. van Poppel

This supplementary material has been provided by the authors to give readers additional information about their work.

Content

Additional Methods 1: Test – Manual – Austrian Balance Check (ABC)

Additional Methods 2: Weight classification

Additional Methods 3: Selection of participants for checking test quality criteria and competitiveness

Table S1. Classification of Austrian Balance Check

Table S2. Overall sample characteristics

Table S3. Differences between regular school classes and elite sports classes

Table S4. Mann–Whitney U-test used to assess differences in classification groups between regular school classes and elite sports classes

Table S5. Gender- and age-specific mean values of raw values of Austrian Balance Check

Table S6. Traditional z-scores and STA nine values

Table S7. Descriptive statistics of the results for checking objectivity

Table S8. Descriptive statistics of the results for checking reliability using the test–retest method

Table S9. Reliability and objectivity of ABC performance in different age groups and separately for boys and girls

Table S10. Descriptive statistics of the results for checking validity using the KINVENT force plate

Table S11. Age-specific data from the validity check using Spearman's correlation coefficient

Table S12. Age- and gender-specific data from the competitiveness review using Spearman's correlation coefficient

Table S13. Comparison of testing times between ABC and BB GMT by using paired t-test

Figure S1. Gender- and age-specific mean values of raw values of Austrian Balance Check

Figure S2. A graphical illustration of the normal distribution using histograms for the classification of the Austrian Balance Check and balancing backwards (German Motor Test)

Additional Methods 1: Test – Manual – Austrian Balance Check (ABC)

The Austrian Balance Check (ABC)

A three-stage field test for children, adolescents and young adults to assess static balance

Test organization

Assessment of static balance using the single leg stand.

Test item

The test task is used to check and assess static balance. Depending on the individual's ability, the test participant completes a maximum of three static balance exercises of increasing difficulty. The aim is to gain as many points as possible.

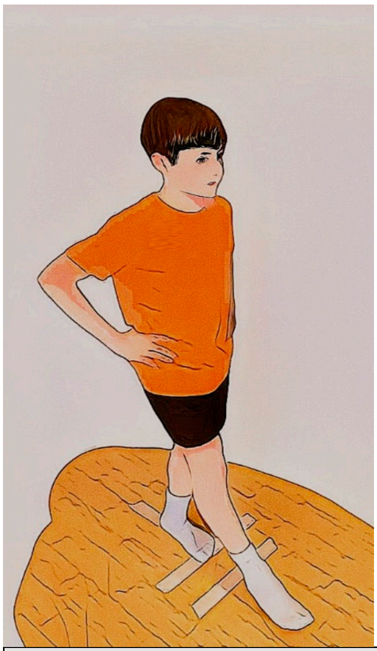
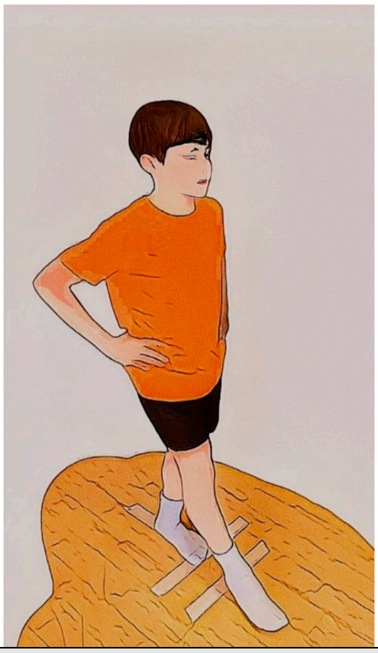
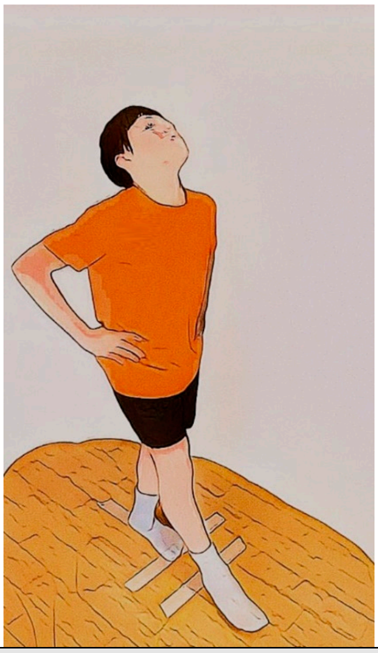

**Test item**		
1	2	3
**Level of difficulty**		
Level 1	Level 2	Level 3
**Tested parameter**		
Static balance		
**Direction of view**		
forward facing	forward facing	upward facing
**Eyes**		
open	open	closed
with visual fixed point	without visual fixed point	without visual fixed point

Test materials

A marking tape and a timer (cell phone or handheld timer) are required.

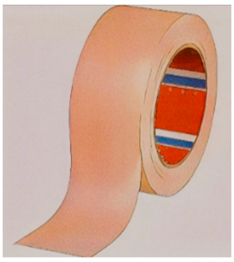

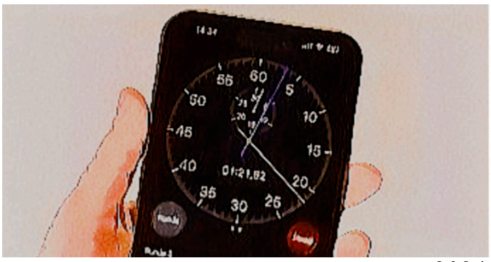

The material required for the three-stage Austrian Balance Check. © JARNIG, 2024.

Test staff

One test administrator can assess up to six test persons at the same time.

Test construction

The construction of a test station: Using marking tape, a T is marked on the floor and another mark is made parallel to the horizontal line (of the T) at a distance of ≈ 5 cm.

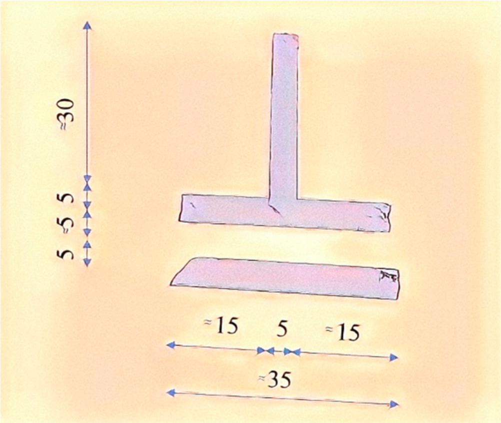

The construction of the three-stage Austrian Balance Check test station (dimensions in cm). © JARNIG, 2024.

In total, six similar test stations are constructed next to each other on the ground; the distance between the test stations should be ≈ 135 cm.

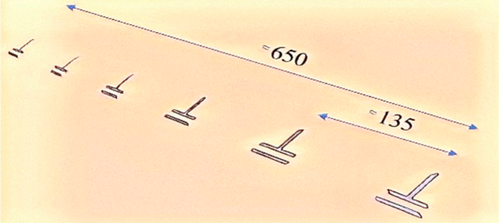

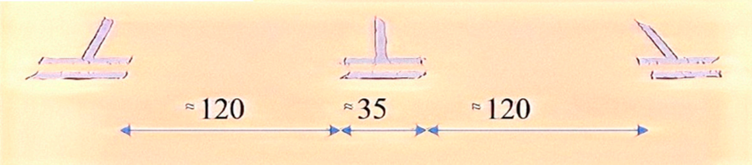

The construction and placement of six test stations of the three-stage Austrian Balance Check (dimensions in cm). © JARNIG, 2024.

Test administrator station: A seat (gym bench, chair) is placed for the test administrator at a distance of at least 4.00 m, centrally opposite the six test stations.

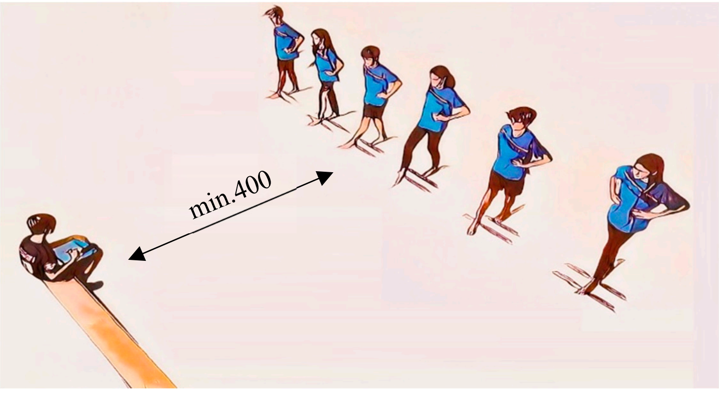

The test administrator station and six test stations of the three-stage Austrian Balance Check (dimensions in cm). © JARNIG, 2024.

Test administration

The test is based on a three-stage test structure with different test items consisting of three increasing levels of difficulty. The test is carried out barefoot or with socks on a non-slip surface.

Test preparation

The test administrator demonstrates the three different test items to the test participants and illustrates execution errors that lead to early termination of an assessment attempt.

The test participants are allocated to a test station and asked to try out the test items with both legs and decide which leg is to be used as the standing leg and which is the playing leg during the test. The choice made must be maintained during all assessment attempts. The test participants have a maximum of 30 seconds to define the standing leg and playing leg for the test items.

Test procedure

For each balance item, a test person has a maximum of two assessment attempts to complete the item successfully. If a participant completes the maximum test duration of difficulty level 1 on the first assessment attempt, the participant has to wait until a possible second assessment attempt at this level has been completed by other test participants. If difficulty level 1 is completed successfully by all test participants in the first assessment attempt, the test continues with the assessment of level 2.
The same conditions apply for difficulty level 2. For difficulty level 3, all participants who have successfully completed difficulty levels 1 and 2 must carry out two assessment attempts (regardless of their performance in the first assessment attempt).
The break between the two assessment attempts must be at least 20 seconds.
The test administrator informs the participants that no information regarding early termination of the assessment attempt will be given during an attempt, as this could influence the test performance of other participants being assessed at the same time. At the end of an assessment round, participants will be informed which ones have to repeat it or have successfully completed it. Participants are instructed to sit down on the floor at the test station if an error in execution has led to early termination of an assessment attempt (*see End of an assessment attempt*).

Level 1: The participant stands with their standing leg on the longitudinal line of the T marker. The toes of the standing leg touch the transverse line of the T mark. Their hands are supported at their hips whilst their thumbs point backwards in this position. The heel of their playing leg is held in the air above the mark that is parallel to the crossbar of the T mark. The direction of view is forward and their eyes stay open during each assessment attempt.

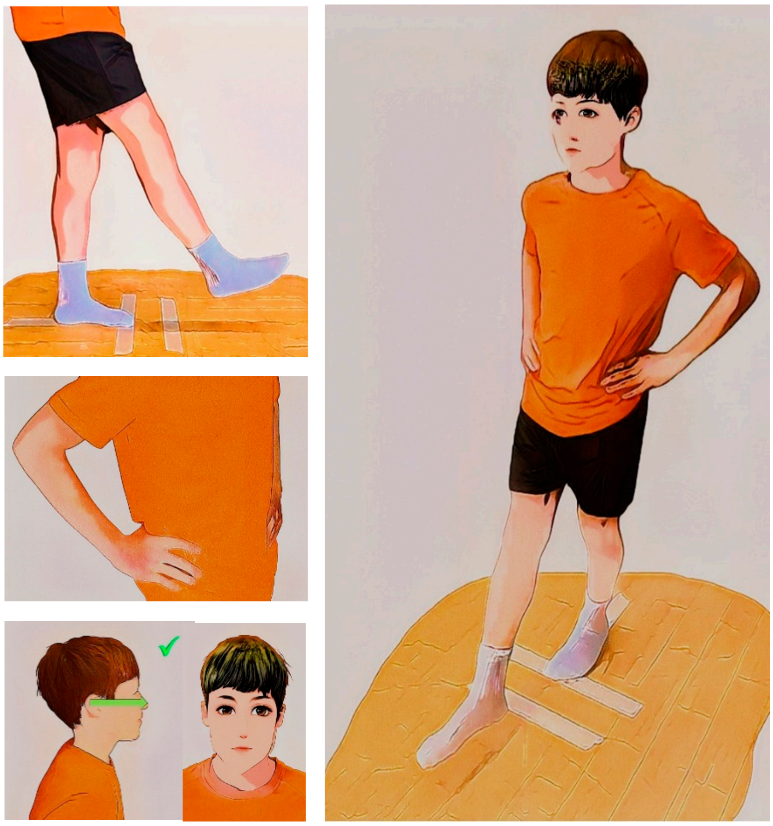

Three-stage Austrian Balance Check—level 1. © JARNIG, 2024.

Level 2: The test is carried out in the same way as for level 1, but the participants' eyes must be closed during the assessment of level 2.

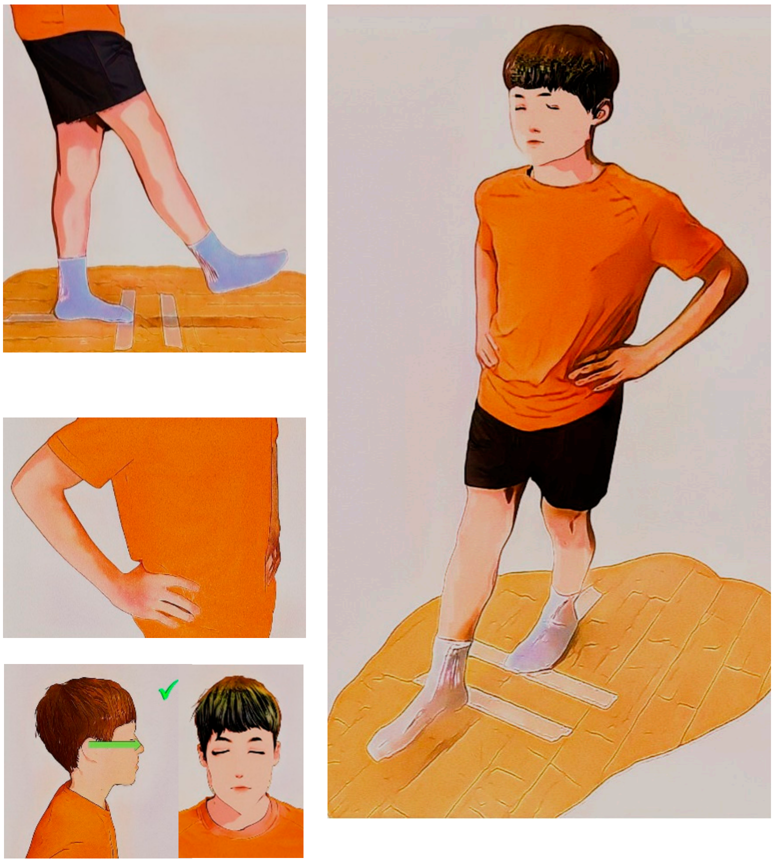

Three-stage Austrian Balance Check—level 2. © JARNIG, 2024.

Level 3: The test is carried out in the same way as for difficulty level 2, with the participants' head facing backwards during testing. Extension of the cervical spine along the sagittal plane is performed. The direction of view is upwards and their eyes must be closed during the assessment. An imaginary line between the end of the earlobe and the eyes is used for control; this should point vertically upwards.

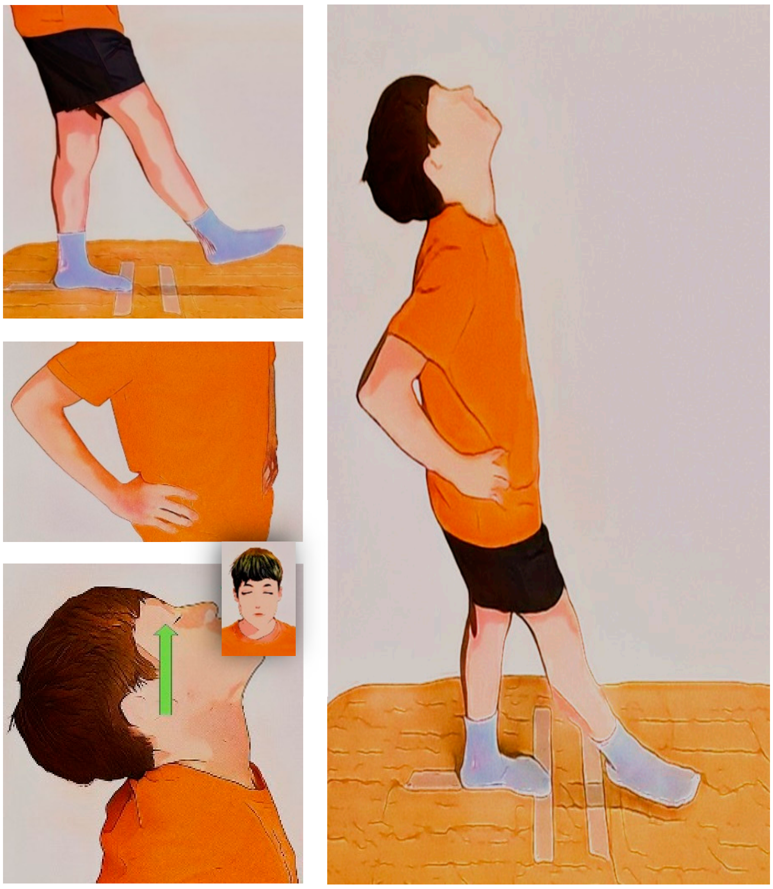

Three-stage Austrian Balance Check—level 3. © JARNIG, 2024.

Test duration, end of assessment attempt and end of test

Test duration—length of different levels

The maximum test duration of an assessment attempt for levels 1 and 2 is 40 seconds (A); for level 3, the maximum possible test duration of an attempt is defined as 20 seconds (B).
The test person must successfully complete a level in order to be allowed to move on to the next level. A level is considered successfully completed if the maximum possible test duration is completed without errors. Each participant has two assessment attempts per level in order to successfully complete it. The test item of difficulty level 3 must be completed twice by all participants who have successfully completed levels 1 and 2, regardless of whether or not the maximum test duration was reached in the first assessment attempt of level 3.

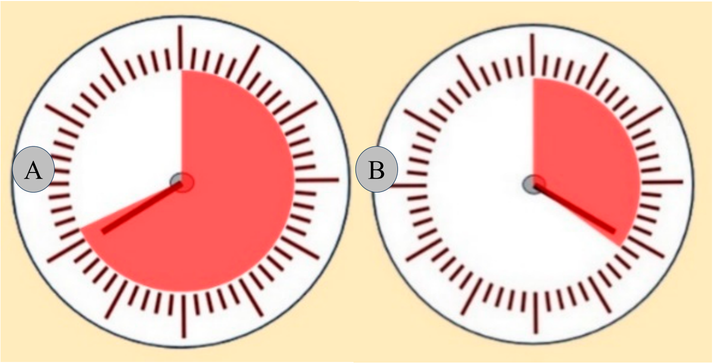

(A) Maximum test duration of levels 1 and 2.
(B) Maximum test duration of level 3. © JARNIG, 2024.

End of an assessment attempt

Execution errors and test rules which lead to the end of an assessment attempt in the different test levels:

Level 1:

(1) One or both hands leave the resting position on the hip or remove contact with the hip.
(2) The standing leg leaves the starting position on the longitudinal line of the T marking.
(3) The heel of the playing leg crosses the transverse line of the T marking.
(4) The playing leg comes into contact with the ground.
(5) The maximum duration of the assessment attempt is reached.

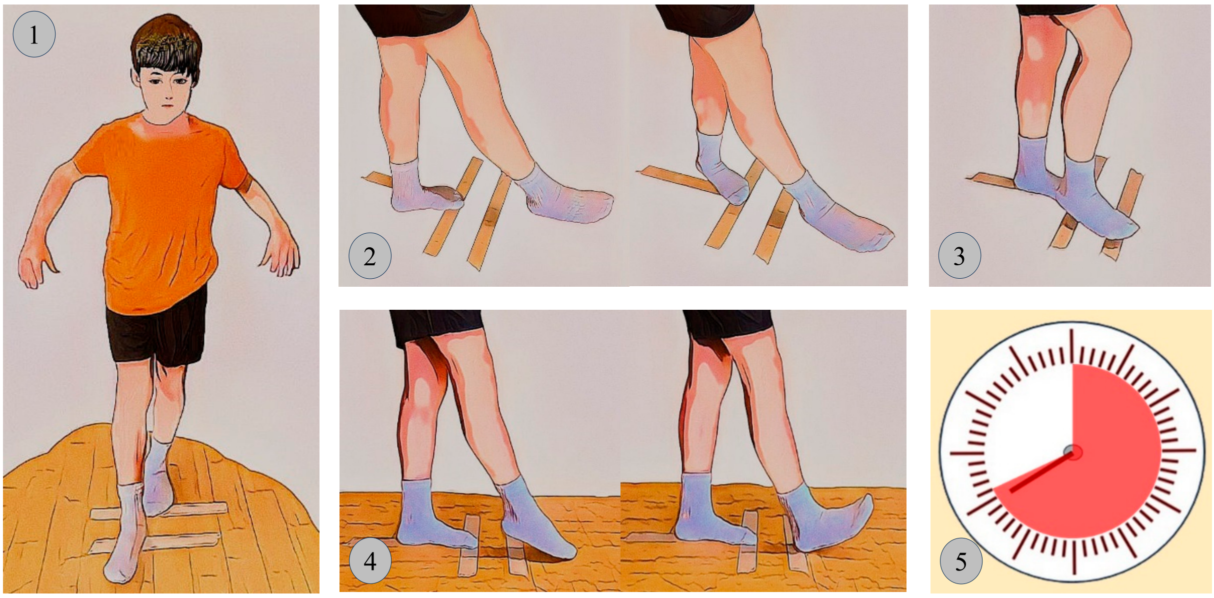

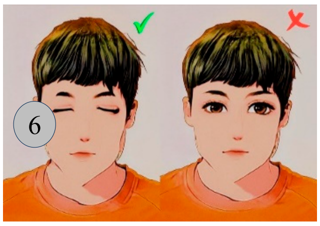

Level 2/3:

(1) – (5) All execution errors and test regulations that lead to the end of an assessment attempt in level 1.
(6) Test participant opening an eye.

Level 3:

(1) – (6) All execution errors and test regulations that lead to the end of an assessment attempt in level 1 and 2 (**attention: ⑤ max. 20 seconds**).
(7) The head leaves the backward-facing position. The imaginary line between the earlobes and closed eyes no longer shows a vertical line.

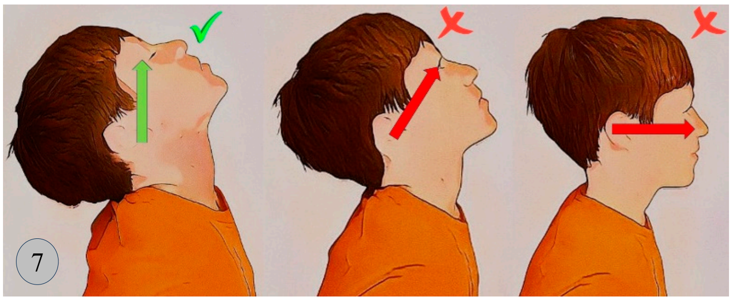

End of a test attempt—level 1 to 3. © JARNIG, 2024.

Test end

If a participant cannot complete the second assessment attempt of level 1 or 2 successfully, this signals the end of the balancing assessment.

If a person reaches level 3, two assessment attempts must be carried out, regardless of whether the maximum test duration was achieved in the first assessment attempt.

Test instructions

The test administrator gives instructions to the participants that are as uniform as possible. There are four different test instructions:

Before testing:

General information 1:	"Please go to test station no. (…), carry out the three different single leg test items with both legs and decide which leg will be used as the standing leg and which as the playing leg when performing the test."
General information 2:	"If a participant makes an execution error which leads to the early termination of an assessment attempt, the participant should sit down on the floor at the test station and wait until all participants have completed the current assessment attempt. An error and the associated end of a participant's assessment attempt will be recorded or documented by me without comment. During an assessment attempt, I will not give any information regarding the early termination of an assessment attempt, as this could influence the performance of other participants. At the end of each assessment attempt, I will let you know who has to repeat it and who has successfully completed it."

Before starting level 1:

"Please go to the starting position, place the selected standing leg on the longitudinal line of the T-marking, keep your hands placed on your hips with your thumbs pointing backwards! The start command will be - Ready, set, go. At 'Ready', move your playing leg above the parallel mark of the horizontal line of the T mark. The command is - Ready, set, go!"

Before starting level 2:

"Please go to the starting position, place the selected standing leg on the longitudinal line of the T-marking, keep your hands placed on your hips with your thumbs pointing backwards! The start command will be - Ready, set, go. At 'Ready', move your playing leg above the parallel mark of the horizontal line of the T mark and close your eyes at 'go'. The command is - Ready, set, go!"

Before starting level 3:

"Please go to the starting position - the hands are held at the hips - thumbs pointing backwards - the head is placed maximally backwards in the neck; the view should be directed vertically upwards! The start command will be - Ready, set, go. At 'Ready', move your playing leg above the parallel mark of the horizontal line of the T mark and close your eyes at 'go'. The command is - Ready, set, go!"

Test documentation/test administration

Recording of data

The assessment sheet, a writing instrument and a stopwatch are required for recording the measurements.

The test administrator takes their place at the test administrator station, keeping an eye on the assessment sheet, the stopwatch and the six test stations at the same time.

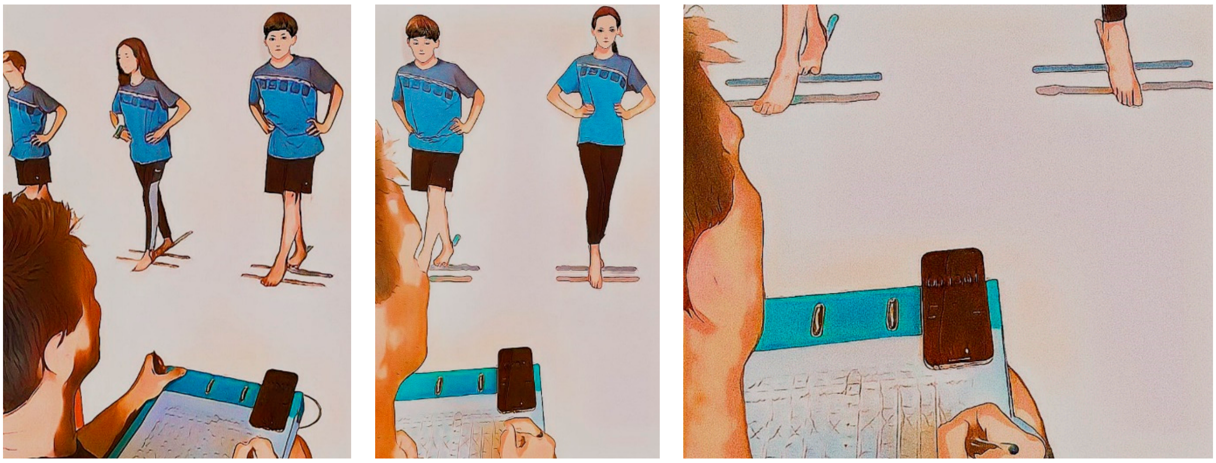

Recording of data—three-stage Austrian Balance Check. © JARNIG, 2024.

The assessment sheet is used to document the time at which a participant terminates an attempt.

Assessment sheet

Using the test assessment sheet, the static balance performance of up to six participants can be documented simultaneously.

The test assessment sheet is filled out by the test administrator in the following order:

General test data: ① Testing date
② Test group

Personal test data: ③ Code of participant
④ Selected standing leg of participant

Test performance: ⑤ The test administrator marks the length of the individual assessment attempts by crossing the appropriate boxes on the assessment sheet. ⑥ An assessment area is available for each assessment attempt. ⑦ In total, there are six assessment areas, two for each of the test items 1 to 3 (level 1-3). ⑧ If an assessment area is not needed by a participant (successful first attempt), this area is clearly crossed out by the test administrator so that there is no confusion as to which area is being tested.

Test result: ⑨ At the bottom of the assessment column for each participant, the test performance can be evaluated directly.

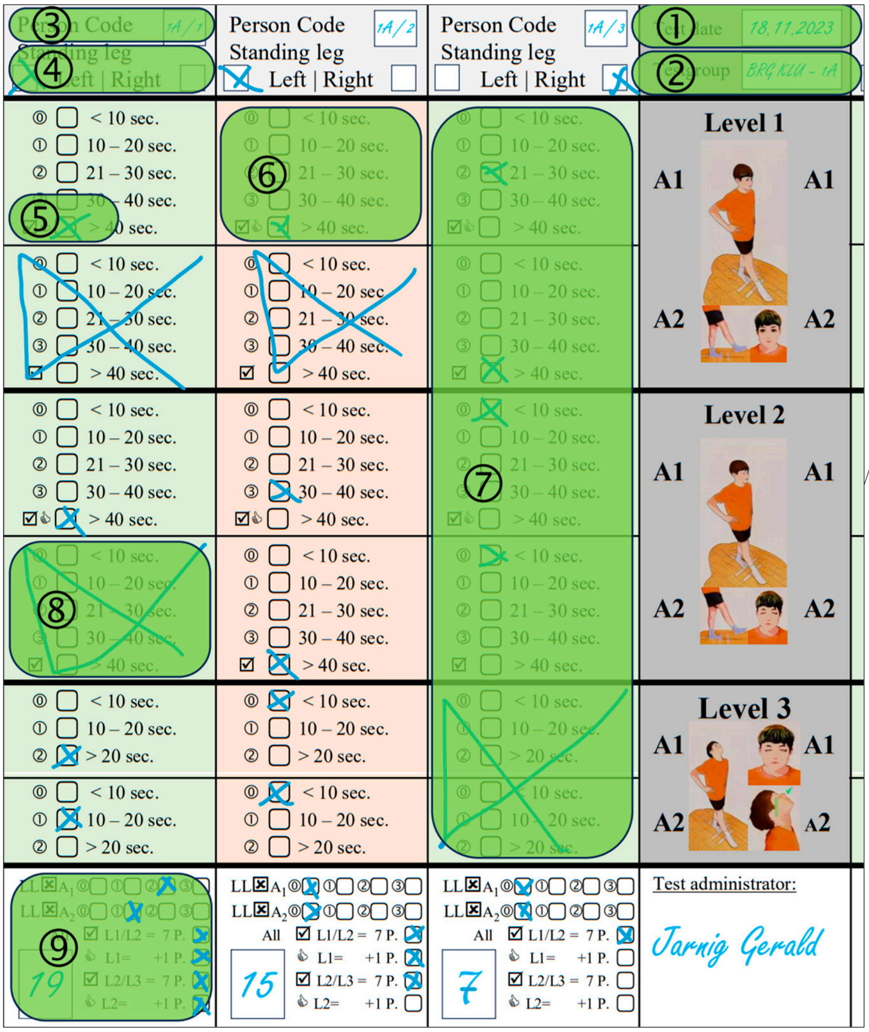

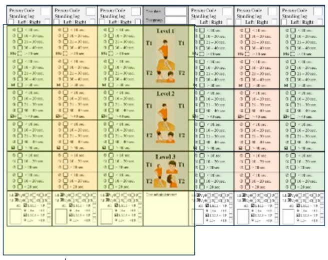

Explanations of the test assessment sheet – Three-stage Austrian Balance Check - © JARNIG, 2024

A1 = first assessment attempt; A2 = second assessment attempt; LL = last level; L1 = level 1; L2 = level 2; L3 = level 3; P. = point/s.

Person Code ☐<br>Standing leg<br>☐ Left | Right ☐	Person Code ☐<br>Standing leg<br>☐ Left | Right ☐	Person Code ☐<br>Standing leg<br>☐ Left | Right ☐	Test date ☐<br>Testgroup ☐	Person Code ☐<br>Standing leg<br>☐ Left | Right ☐	Person Code ☐<br>Standing leg<br>☐ Left | Right ☐	Person Code ☐<br>Standing leg<br>☐ Left | Right ☐
⓪ ☐ < 10 sec.<br>① ☐ 10 – 20 sec.<br>② ☐ 21 – 30 sec.<br>③ ☐ 30 – 40 sec.<br>☑ ☐ > 40 sec.	⓪ ☐ < 10 sec.<br>① ☐ 10 – 20 sec.<br>② ☐ 21 – 30 sec.<br>③ ☐ 30 – 40 sec.<br>☑ ☐ > 40 sec.	⓪ ☐ < 10 sec.<br>① ☐ 10 – 20 sec.<br>② ☐ 21 – 30 sec.<br>③ ☐ 30 – 40 sec.<br>☑ ☐ > 40 sec.	**Level 1**<br>A1 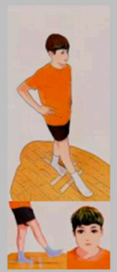 A1	⓪ ☐ < 10 sec.<br>① ☐ 10 – 20 sec.<br>② ☐ 21 – 30 sec.<br>③ ☐ 30 – 40 sec.<br>☑ ☐ > 40 sec.	⓪ ☐ < 10 sec.<br>① ☐ 10 – 20 sec.<br>② ☐ 21 – 30 sec.<br>③ ☐ 30 – 40 sec.<br>☑ ☐ > 40 sec.	⓪ ☐ < 10 sec.<br>① ☐ 10 – 20 sec.<br>② ☐ 21 – 30 sec.<br>③ ☐ 30 – 40 sec.<br>☑ ☐ > 40 sec.
⓪ ☐ < 10 sec.<br>① ☐ 10 – 20 sec.<br>② ☐ 21 – 30 sec.<br>③ ☐ 30 – 40 sec.<br>☑ ☐ > 40 sec.	⓪ ☐ < 10 sec.<br>① ☐ 10 – 20 sec.<br>② ☐ 21 – 30 sec.<br>③ ☐ 30 – 40 sec.<br>☑ ☐ > 40 sec.	⓪ ☐ < 10 sec.<br>① ☐ 10 – 20 sec.<br>② ☐ 21 – 30 sec.<br>③ ☐ 30 – 40 sec.<br>☑ ☐ > 40 sec.	A2 A2	⓪ ☐ < 10 sec.<br>① ☐ 10 – 20 sec.<br>② ☐ 21 – 30 sec.<br>③ ☐ 30 – 40 sec.<br>☑ ☐ > 40 sec.	⓪ ☐ < 10 sec.<br>① ☐ 10 – 20 sec.<br>② ☐ 21 – 30 sec.<br>③ ☐ 30 – 40 sec.<br>☑ ☐ > 40 sec.	⓪ ☐ < 10 sec.<br>① ☐ 10 – 20 sec.<br>② ☐ 21 – 30 sec.<br>③ ☐ 30 – 40 sec.<br>☑ ☐ > 40 sec.
⓪ ☐ < 10 sec.<br>① ☐ 10 – 20 sec.<br>② ☐ 21 – 30 sec.<br>③ ☐ 30 – 40 sec.<br>☑ ☐ > 40 sec.	⓪ ☐ < 10 sec.<br>① ☐ 10 – 20 sec.<br>② ☐ 21 – 30 sec.<br>③ ☐ 30 – 40 sec.<br>☑ ☐ > 40 sec.	⓪ ☐ < 10 sec.<br>① ☐ 10 – 20 sec.<br>② ☐ 21 – 30 sec.<br>③ ☐ 30 – 40 sec.<br>☑ ☐ > 40 sec.	**Level 2**<br>A1 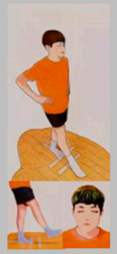 A1	⓪ ☐ < 10 sec.<br>① ☐ 10 – 20 sec.<br>② ☐ 21 – 30 sec.<br>③ ☐ 30 – 40 sec.<br>☑ ☐ > 40 sec.	⓪ ☐ < 10 sec.<br>① ☐ 10 – 20 sec.<br>② ☐ 21 – 30 sec.<br>③ ☐ 30 – 40 sec.<br>☑ ☐ > 40 sec.	⓪ ☐ < 10 sec.<br>① ☐ 10 – 20 sec.<br>② ☐ 21 – 30 sec.<br>③ ☐ 30 – 40 sec.<br>☑ ☐ > 40 sec.
⓪ ☐ < 10 sec.<br>① ☐ 10 – 20 sec.<br>② ☐ 21 – 30 sec.<br>③ ☐ 30 – 40 sec.<br>☑ ☐ > 40 sec.	⓪ ☐ < 10 sec.<br>① ☐ 10 – 20 sec.<br>② ☐ 21 – 30 sec.<br>③ ☐ 30 – 40 sec.<br>☑ ☐ > 40 sec.	⓪ ☐ < 10 sec.<br>① ☐ 10 – 20 sec.<br>② ☐ 21 – 30 sec.<br>③ ☐ 30 – 40 sec.<br>☑ ☐ > 40 sec.	A2 A2	⓪ ☐ < 10 sec.<br>① ☐ 10 – 20 sec.<br>② ☐ 21 – 30 sec.<br>③ ☐ 30 – 40 sec.<br>☑ ☐ > 40 sec.	⓪ ☐ < 10 sec.<br>① ☐ 10 – 20 sec.<br>② ☐ 21 – 30 sec.<br>③ ☐ 30 – 40 sec.<br>☑ ☐ > 40 sec.	⓪ ☐ < 10 sec.<br>① ☐ 10 – 20 sec.<br>② ☐ 21 – 30 sec.<br>③ ☐ 30 – 40 sec.<br>☑ ☐ > 40 sec.
⓪ ☐ < 10 sec.<br>① ☐ 10 – 20 sec.<br>② ☐ > 20 sec.	⓪ ☐ < 10 sec.<br>① ☐ 10 – 20 sec.<br>② ☐ > 20 sec.	⓪ ☐ < 10 sec.<br>① ☐ 10 – 20 sec.<br>② ☐ > 20 sec.	**Level 3**<br>A1 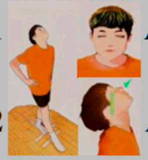 A1	⓪ ☐ < 10 sec.<br>① ☐ 10 – 20 sec.<br>② ☐ > 20 sec.	⓪ ☐ < 10 sec.<br>① ☐ 10 – 20 sec.<br>② ☐ > 20 sec.	⓪ ☐ < 10 sec.<br>① ☐ 10 – 20 sec.<br>② ☐ > 20 sec.
⓪ ☐ < 10 sec.<br>① ☐ 10 – 20 sec.<br>② ☐ > 20 sec.	⓪ ☐ < 10 sec.<br>① ☐ 10 – 20 sec.<br>② ☐ > 20 sec.	⓪ ☐ < 10 sec.<br>① ☐ 10 – 20 sec.<br>② ☐ > 20 sec.	A2 A2	⓪ ☐ < 10 sec.<br>① ☐ 10 – 20 sec.<br>② ☐ > 20 sec.	⓪ ☐ < 10 sec.<br>① ☐ 10 – 20 sec.<br>② ☐ > 20 sec.	⓪ ☐ < 10 sec.<br>① ☐ 10 – 20 sec.<br>② ☐ > 20 sec.
LL☒A1⓪☐ ①☐ ②☐ ③☐<br>LL☒A2⓪☐ ①☐ ②☐ ③☐<br>All ☐ ☑ L1/L2 = 7 P. ☐<br>L1= +1 P. ☐<br>☑ L2/L3 = 7 P. ☐<br>L2= +1 P. ☐	LL☒A1⓪☐ ①☐ ②☐ ③☐<br>LL☒A2⓪☐ ①☐ ②☐ ③☐<br>All ☐ ☑ L1/L2 = 7 P. ☐<br>L1= +1 P. ☐<br>☑ L2/L3 = 7 P. ☐<br>L2= +1 P. ☐	LL☒A1⓪☐ ①☐ ②☐ ③☐<br>LL☒A2⓪☐ ①☐ ②☐ ③☐<br>All ☐ ☑ L1/L2 = 7 P. ☐<br>L1= +1 P. ☐<br>☑ L2/L3 = 7 P. ☐<br>L2= +1 P. ☐	Test administrator:	LL☒A1⓪☐ ①☐ ②☐ ③☐<br>LL☒A2⓪☐ ①☐ ②☐ ③☐<br>All ☐ ☑ L1/L2 = 7 P. ☐<br>L1= +1 P. ☐<br>☑ L2/L3 = 7 P. ☐<br>L2= +1 P. ☐	LL☒A1⓪☐ ①☐ ②☐ ③☐<br>LL☒A2⓪☐ ①☐ ②☐ ③☐<br>All ☐ ☑ L1/L2 = 7 P. ☐<br>L1= +1 P. ☐<br>☑ L2/L3 = 7 P. ☐<br>L2= +1 P. ☐	LL☒A1⓪☐ ①☐ ②☐ ③☐<br>LL☒A2⓪☐ ①☐ ②☐ ③☐<br>All ☐ ☑ L1/L2 = 7 P. ☐<br>L1= +1 P. ☐<br>☑ L2/L3 = 7 P. ☐<br>L2= +1 P. ☐

Assessment sheet designed for simultaneous testing of up to 6 participants. Three-stage Austrian Balance Check.

© JARNIG, 2024.

Test evaluation

The performance of the participants at the difficulty levels achieved in the test of balance is summarized at the bottom of the assessment column on the assessment sheet.

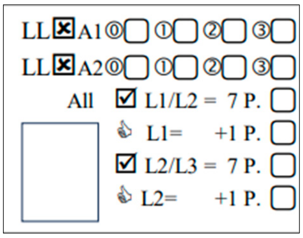

The test evaluation area on the assessment sheet. Three-stage Austrian Balance Check. © JARNIG, 2024.

Points are collected for each assessment attempt, depending on the time a participant takes to perform the test position correctly. Every 10 correctly completed seconds, a participant collects one point for the overall result (**attention:** only in the last level achieved).
Additional points are given for successfully completing the maximum test duration of test item 1 (difficulty level 1 = +7 points) or test item 2 (difficulty level 2 = +7 points).
If a participant achieves the maximum test duration for the test item in test level 1 (difficulty level 1 = + 1 point) and/or the test item in test level 2 (difficulty level 2 = + 1 point) on the first assessment attempt, a further additional point is collected in each case.

For each participant, six different evaluation steps are carried out to calculate the points of the overall result:

1) LL☒A1 ⓪☐ ①☐ ②☐ ③☐: The first assessment attempt of the highest level achieved is evaluated. The participant collects between 0 and 3 points for the overall result.
2) LL☒A2 ⓪☐ ①☐ ②☐ ③☐: The second assessment attempt of the highest level achieved is evaluated. The participant collects between 0 and 3 points for the overall result.
3) ☑ L1/L2 = 7 P. ☐ : For successfully completing the maximum test duration (>40 sec.) of level 1, this person collects +7 points for the overall result.
4) 👍 L1= +1 P. ☐: If the maximum test duration (>40 sec.) of level 1 is reached in the first assessment attempt, the participant collects an additional point for the overall result.
5) ☑ L2/L3 = 7 P. ☐ : For successfully completing the maximum test duration (>40 sec.) of level 2, this person collects +7 points for the overall result.
6) 👍 L2= +1 P. ☐: If the maximum test duration (>40 sec.) of level 2 is reached in the first assessment attempt, the participant collects an additional point for the overall result.

Real-life examples

Participants who do not complete level 1 correctly

Test procedure

First, the four data columns (test date, test group, personal code and standing leg) are filled in in the header. Ⓐ
The participant loses the correct body position required in level 1 on the first attempt after 33 seconds, noted on the assessment sheet by the test leader. Ⓑ
When trying again, the subject is able to maintain the correct body position for 25 seconds. Ⓒ
Due to the unsuccessful completion of level 1, the ABC ends at this point and the assessment areas for levels 2 and 3 are crossed out. Ⓓ

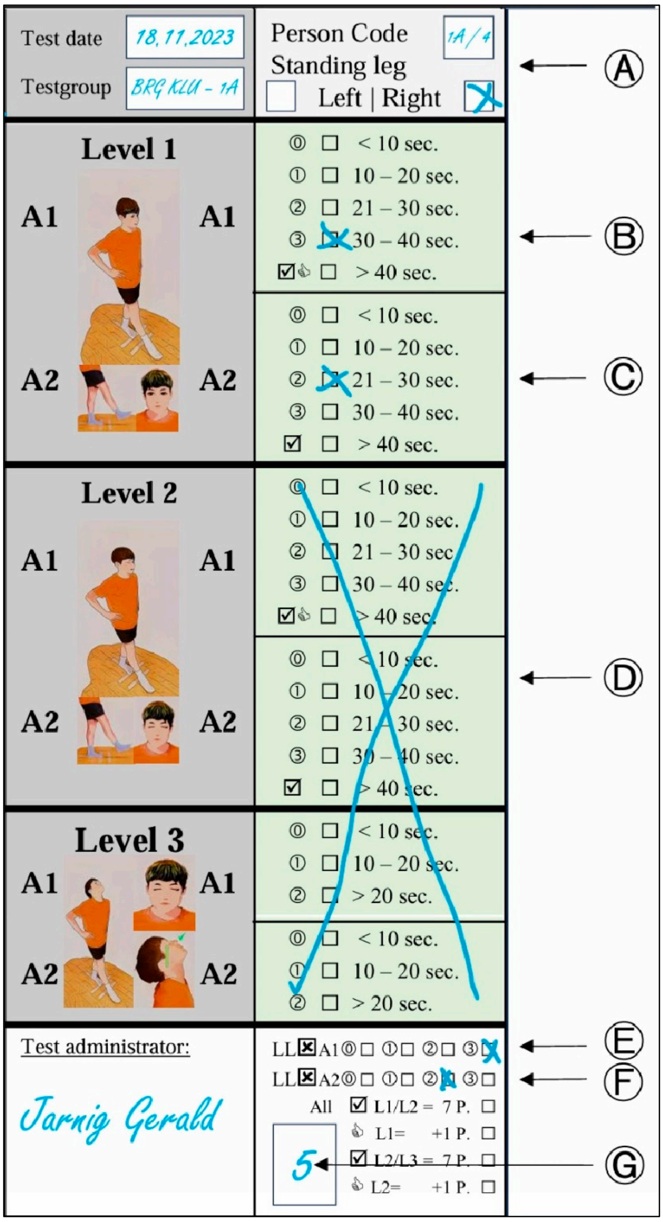

Test analysis:
(at the end of the assessment column)

Only level 1 was carried out, so the points from the two attempts in level 1 are used to calculate the overall result. A time of 33 seconds was achieved in the first attempt for level 1, and for every 10 seconds that the correct body position was maintained, the participant collected 1 point for the overall assessment, so 3 points were collected in this attempt. Ⓔ
In the second attempt, the participant was able to maintain the correct body position for 25 seconds and collected 2 points for doing so. Ⓕ
The points from attempts 1 and 2 are summed up and result in a total of 5 points as the participant's overall assessment score in the ABC. Ⓖ

Participants who do not complete level 2 correctly

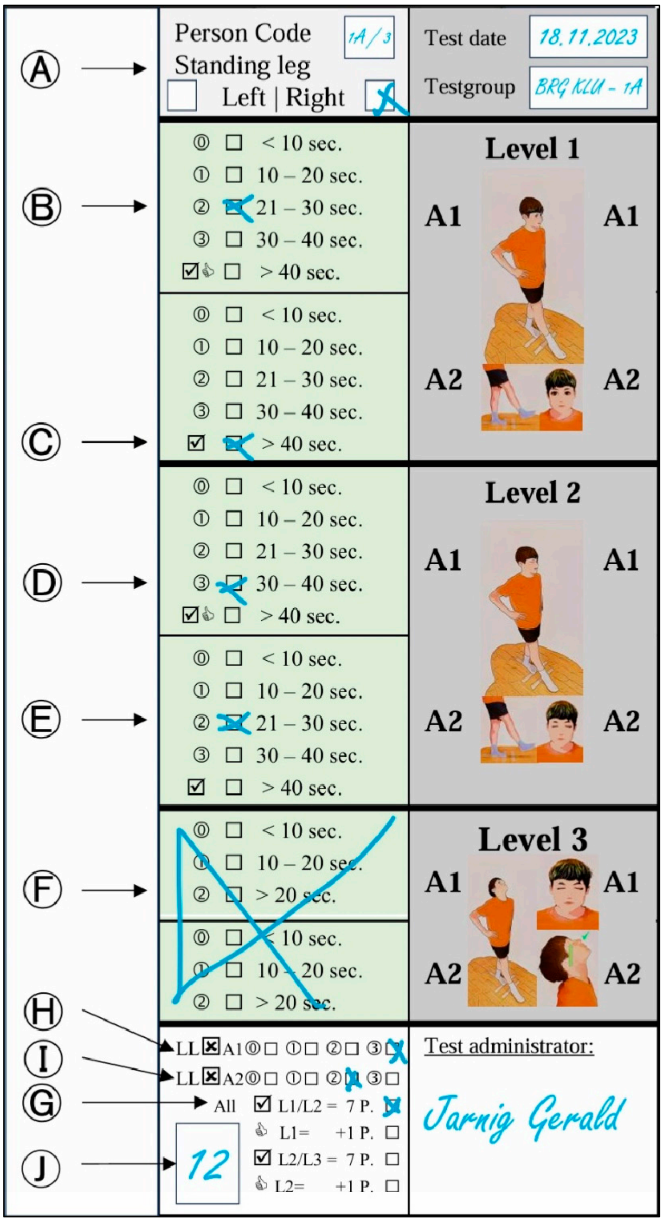

Test procedure

First, the four data columns (test date, test group, personal code and standing leg) are filled in in the header. Ⓐ
The participant loses the correct body position required in level 1 on the first attempt after 34 seconds, noted on the assessment sheet by the test leader. Ⓑ
In the second attempt for level 1, the participant can maintain the correct body position for > 40 seconds. Ⓒ
Thus, level 1 is successfully completed and the participant starts their first attempt for level 2 and is able to maintain the required body position for 33 seconds; this is recorded on the assessment sheet by the test leader. Ⓓ
The second time they try to complete level 2, the participant is able to maintain the correct body position for 27 seconds. Ⓔ
As level 2 is not successfully completed, the ABC ends at this point and the assessment area for level 3 is crossed out. Ⓕ

Test analysis:
(at the end of the assessment column)

Level 1 was successfully completed, resulting in a total of 7 points for the overall assessment. Ⓖ
Level 2 was not successfully completed, and therefore, the points from the two attempts for level 2 are calculated. During the first attempt for level 2, a time of 33 seconds was reached. For every 10 seconds that the correct body position was maintained, the participant collected 1 point toward their overall assessment score, so 3 points were collected during this attempt. Ⓗ
In the second attempt, the participant was able to maintain the correct body position for 27 seconds, collecting 2 points. Ⓘ
All points collected result in a total of 12 points (7 points from level 1 and 5 points from level 2). Ⓙ

Participants who reach level 3

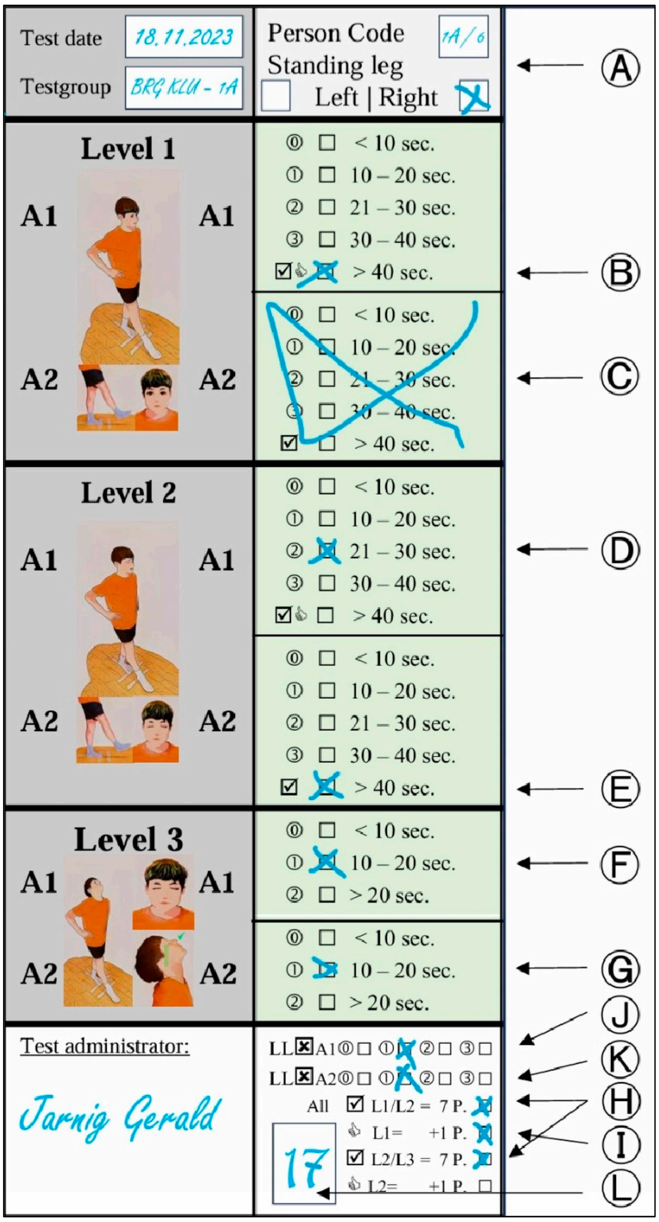

Test procedure

First, the four data columns (test date, test group, personal code and standing leg) are filled in in the header. Ⓐ
The participant successfully completes level 1 in their first attempt; this is recorded on the assessment sheet by the test leader. Ⓑ The second evaluation area in level 1 is then crossed out. Ⓒ
This means that level 1 has been successfully completed and the participant starts their first attempt for level 2. During this attempt, the participant can maintain the required body position for 23 seconds, which is documented on the assessment sheet by the test leader. Ⓓ
In their second attempt for level 2, the participant is able to maintain the correct body position for > 40 seconds. This means that level 2 has been successfully completed. Ⓔ
The participant fails to hold the correct body position required for level 3 in their first attempt after 13 seconds, which is recorded by the test leader on the assessment sheet. Ⓕ
In their second attempt for level 3, the participant can maintain the correct body position for 15 seconds, which is recorded on the assessment sheet by the test administrator. Ⓖ The ABC ends at this point.

Test analysis:
(at the end of the assessment column)

Levels 1 and 2 were successfully completed, thereby collecting 7 points per level for the overall assessment. Ⓗ Additionally, level 1 was successfully completed in the first attempt, thereby collecting 1 additional point for the overall assessment score. Ⓘ
The individual points from the two attempts for level 3 are calculated. In the first attempt for level 3, a time of 13 seconds was achieved. For every 10 seconds that the correct body position was maintained, the participant collected 1 point toward the overall assessment score. Ⓙ
During their second attempt, the participant was able to maintain the correct body position for 15 seconds and collected 1 point for doing so. Ⓚ

All points collected result in a total of 17 points (2 x 7 points for successfully completing levels 1 and 2; 1 additional point from level 1 for successfully completing the test on the first attempt; and 2 points from level 3). Ⓛ

Performance assessment/reference values

Two different assessment models are available for the performance assessment, which allow for a rough classification into three performance categories (low, average and high balance performance) and a more detailed classification into nine performance categories.

The Categories of the Austrian Balance Check:

The reference values are applicable for children, adolescents and young adults of school age and above; these values are not suitable for kindergarten children and children in pre-school classes.

Variable	Gender	Age group	Total points achieved (0 to 20) in the Austrian Balance Check																				
			0	1	2	3	4	5	6	7	8	9	10	11	12	13	14	15	16	17	18	19	20
Allocation of the total points to the performance categories	Male	≤ 7	1	2	3	3	4	5	5	6	6	7	8	8	9	9	9	9	9	9	9	9	9
		8 to 9	1	2	2	3	3	4	4	5	5	6	6	6	7	7	8	8	9	9	9	9	9
		10 to 11	1	1	2	2	2	3	3	4	4	5	5	6	6	6	7	7	8	8	9	9	9
		12 to 13	1	1	1	1	1	2	2	3	3	4	4	5	5	6	7	7	8	8	9	9	9
		14 to 15	1	1	1	1	1	1	2	2	3	4	4	5	5	6	6	7	7	8	9	9	9
		16 to 17	1	1	1	1	1	1	1	2	2	3	3	4	5	5	6	7	7	8	9	9	9
		18 to 19	1	1	1	1	1	1	1	1	2	2	3	4	4	5	6	6	7	8	8	9	9
	Female	≤ 7	1	2	2	3	3	4	4	5	5	6	6	7	7	8	8	9	9	9	9	9	9
		8 to 9	1	1	2	2	3	3	4	4	5	5	6	6	7	7	8	8	9	9	9	9	9
		10 to 11	1	1	1	1	1	1	2	3	3	4	5	5	6	7	8	8	9	9	9	9	9
		12 to 13	1	1	1	1	1	1	2	2	3	3	4	5	5	6	6	7	8	8	9	9	9
		14 to 15	1	1	1	1	1	1	1	2	3	3	4	4	5	6	6	7	8	8	9	9	9
		16 to 17	1	1	1	1	1	1	1	2	2	3	3	4	5	5	6	6	7	7	8	8	9
		18 to 19	1	1	1	1	1	1	1	1	2	2	3	4	4	5	5	6	6	7	8	8	9

Categories of classification

Low balance performance		Average balance performance		High balance performance	
1	Poor balance	4	Below-average balance	7	Good balance
2	Very weak balance	5	Average balance	8	Very good balance
3	Weak balance	6	Above-average balance	9	Excellent balance

Potential errors

Possible sources of error that may occur:

- Changing the participants' standing position between test attempts.
- Mixing up the assessment column or assessment line on the assessment sheet during the test procedure.
- The test being carried out while wearing sports shoes.
- A participant being disturbed by another participant while carrying out the test.

Other information

The test is carried out in the sports hall, an exercise room, or a comparable space. If it is possible for two test administrators to carry out the test, 12 test stations can be set up.

Test Box

WHAT IS ALREADY KNOWN ON THIS TOPIC

⇒ Balance is a good indicator of overall physical fitness and intellectual fitness.
⇒ In many international fitness test batteries in the field, the assessment of balance is not taken into account.

WHAT THIS STUDY ADDS

⇒ The Austrian Balance Check makes it possible to evaluate the static balance performance of large groups within a short time and without the need for cost-intensive test material.
⇒ The test quality criteria of reliability, objectivity, validity, standardization/categorization, competitiveness and economy are analyzed and evaluated.
⇒ This study provides the first gender- and age-specific reference values for the classification of the measured balance performances of the three-stage Austrian Balance Check.
⇒ The supplementary material includes a detailed test manual which can be used to carry out the test without any specific prior knowledge.

HOW THIS STUDY MIGHT AFFECT RESEARCH, PRACTICE OR POLICY

⇒ The Austrian Balance Check makes it easy to assess balance performance in schools, health centers, sports clubs, companies and private households.
⇒ By saving time, increasing the associated cost efficiency, reducing tester tiredness and increasing test accessibility, improved test acceptance should lead to increased use of balance assessments in international test batteries.
⇒ The newly created option of testing static balance in the field can be used in cross-sectional and long-term studies.
⇒ The Austrian Balance Check can play a supporting role in assessing children's 'school readiness'.

Additional Methods 2: Weight classification

National reference values were used for the standardization of the BMI and the classification of weight. [1]
The national reference data were expressed in BMI centile curves (i.e., equicurves, called EQUI BMI in this report) [1]. The absolute BMI values were converted to EQUI BMI values according to the procedure described in Mayer et al. [1] (based on Cole et al. [2]). The EQUI BMI curves were used to project the actual BMI cut-off values at 18 years of age, allowing us to categorize the children's weight into five categories (underweight < 18.5 kg/m2, normal weight = 18.5 to 25.0 kg/m2, overweight ≥ 25.0 kg/m2, obese ≥ 30.0 kg/m2, morbid obesity ≥ 35.0 kg/m2). For participants aged > 18 years, the crude BMI was used to assign a weight classification.

Additional Methods 3: Selection of participants for checking test quality criteria and competitiveness

The validity test on the force plate showed that participants were often unable to hold the balance position three times in a row (with a break of 7 seconds) without touching the ground with their playing leg when performing the tests with their eyes closed. As the ABC evaluates performance with eyes open and eyes closed as a whole, only data from participants who were able to complete the entire test on the force plate without errors (without touching the ground) were included in the calculations. Touching the ground one or more times during the test was defined as errors and the participants were excluded from the validity analyses, as it was not technically possible to eliminate these execution errors (ground contacts) from the remaining data.

Table S1. Classification of Austrian Balance Check.

Classification of STA9 values into a nine-point rating scale			
**STA9 values**	**ABC Category**		
<2.0	1	Low b.	No b.
2.0 to 3.0	2		poor b.
3.0 to 4.0	3		Weak b.
4.0 to 5.0	4	Average b.	Below-average b.
5.0 to 6.0	5		Average b.
6.0 to 7.0	6		Above-average b.
7.0 to 8.0	7	High b.	Good b.
8.0 to 9.0	8		Very good b.
≥9.0	9		Excellent b.

STA9 = Stanine values, short for "standard nine", are a method of scaling test results on a nine-point standard scale. The Z-scores of the total achievable points were converted to a nine-point scale (STA9) with a mean of 5 and a standard deviation of 2; ABC = Austrian Balance Check; b = balance.

Table S2. Overall sample characteristics.

Variable				Regular school class			Elite sports school class		
				**All**	**Boys**	**Girls**	**All**	**Boys**	**Girls**
**N**				609	286	323	396	308	88
**Age, years**				13.0 (3.7)	12.6 (3.7)	13.4 (3.7)	13.6 (2.6)	13.7 (2.6)	13.2 (2.6)
**Anthropometric data**	**Weight, kg**			50.9 (19.2)	51.7 (20.6)	50.2 (17.8)	52.9 (16.0)	54.6 (16.3)	46.9 (13.3)
	**Height, cm**			155.5 (18.5)	157.4 (21.3)	153.9 (15.3)	162.6 (15.8)	164.4 (16.0)	156.3 (13.3)
	**BMI, kg/m^2**			20.3 (4.5)	19.9 (4.1)	20.6 (4.8)	19.5 (2.9)	19.7 (2.8)	18.8 (2.8)
	**EQUI BMI$_{AUT}$**			22.9 (4.1)	23.2 (4.0)	22.7 (4.2)	21.9 (2.3)	22.2 (2.2)	20.9 (2.1)
	**AUT weight classification, No. (%)**	Underweight		46 (7.6)	11 (3.8)	35 (10.8)	21 (5.3)	10 (3.2)	11 (12.5)
		Normal weight		420 (69.0)	205 (71.7)	215 (66.6)	336 (84.8)	264 (85.7)	72 (81.8)
		Overweight		100 (16.4)	48 (16.8)	52 (16.1)	39 (9.8)	34 (11.0)	5 (5.7)
		Obesity		34 (5.6)	18 (6.3)	16 (5.0)	0 (0.0)	0 (0.0)	0 (0.0)
		Morbid obesity		9 (1.5)	4 (1.4)	5 (1.5)	0 (0.0)	0 (0.0)	0 (0.0)
**Balance performances**	**ABC**	**ABC, rs**		9.78 (4.17)	9.13 (4.37)	10.35 (3.91)	13.04 (3.61)	13.04 (3.56)	13.05 (3.82)
	**Test of reliability**	**N**		342	161	181	139	104	35
		**Age, years**		13.3 (3.8)	12.9 (3.9)	13.6 (3.7)	13.2 (1.8)	13.2 (1.8)	13.3 (1.7)
		**ABC 1, rs**		9.74 (4.39)	9.07 (4.70)	10.33 (4.03)	12.37 (3.50)	12.25 (3.42)	12.74 (3.75)
		**ABC 2, rs**		9.76 (4.50)	8.88 (4.57)	10.55 (4.31)	12.79 (3.40)	12.70 (3.36)	13.06 (3.65)
	**Test of objectivity**	**N**		210	110	100	73	52	21
		**Age, years**		12.0 (3.9)	11.5 (3.7)	12.7 (4.0)	13.5 (0.8)	13.5 (0.8)	13.6 (0.8)
		**ABC R1 , rs**		8.56 (4.61)	7.96 (4.79)	9.22 (4.33)	11.78 (3.46)	11.75 (3.58)	11.86 (3.23)
		**ABC R2, rs**		8.54 (4.62)	7.96 (4.81)	9.17 (4.34)	11.71 (3.47)	11.69 (3.59)	11.76 (3.25)
	**Test of validity**	**N**		163	67	96	ND	ND	ND
		**Age, years**		14.3 (3.0)	13.9 (2.9)	14.6 (3.0)	ND	ND	ND
		**ABC, rs**		12.08 (3.52)	10.66 (2.90)	13.07 (3.58)	ND	ND	ND
		**FP SLS, rs**	**COP Su, mm^2**	3463 (1451)	4025 (1726)	3071 (1069)	ND	ND	ND
			**COP Mv, mm/s**	152.9 (44.7)	176.8 (50.7)	136.2 (30.7)	ND	ND	ND
			**COP Pl, mm**	776 (228)	899 (258)	690 (156)	ND	ND	ND
	**Competitiveness with established balance field test**	**N**		458	223	235	279	214	65
		**Age, years**		12.2 (3.5)	12.1 (3.6)	12.4 (3.5)	13.3 (2.4)	13.4 (2.4)	13.3 (2.5)
		**ABC, rs**		9.41 (4.20)	8.85 (4.47)	9.94 (3.85)	13.01 (3.64)	12.88 (3.64)	13.4 (3.6)
		**Time ABC - min.**		1.15 (0.09)	1.16 (0.09)	1.14 (0.09)	1.07 (0.07)	1.07 (0.07)	1.07 (0.07)
		**BB GMT, rs**		29.3 (11.5)	27.0 (12.3)	31.4 (10.3)	40.7 (7.4)	39.8 (7.6)	43.8 (5.8)
		**Time BB GMT, min.**		2.43 (0.23)	2.45 (0.24)	2.41 (0.22)	2.36 (0.19)	2.37 (0.19)	2.31 (0.19)

N = study population. Data are No. (%) or mean (SD). No. = number; % = percent; SD = standard deviation; kg = kilogram; cm = centimeter; BMI = body mass index; kg/m2 = kilogram/meter squared; EQUI BMI$_{AUT}$ = equivalent BMI based on Austrian reference centile curves passing through adult BMI values; ABC = Austrian Balance Check; T = measurement time point; rs = raw score; FB = force plate; SLS = single-leg stand; COP = center of pressure; Su = surface; Mv = medium velocity; Pl = path length; BB = balancing backwards; GMT = German Motor Test; mm^2 = square millimeter; mm/s = millimeters per second; min = minute; ND = no data.

Table S3. Differences between regular school classes and elite sports classes.

Variable	Regular school class [N=609]	Elite sports school class [N=396]	Regular school class vs. elite sports school class		
			t	*P* value	p-lvl
**Age, years**	13.0 (3.7)	13.6 (2.6)	2.918	0.002	**
**Weight, kg**	50.9 (19.2)	52.9 (16.0)	1.717	0.09	
**Height, cm**	155.5 (18.5)	162.6 (15.8)	6.232	<.001	***
**BMI, kg/m2**	20.3 (4.5)	19.5 (2.9)	3.042	0.002	**
**EQUI BMI$_{AUT}$, mean (SD)**	22.9 (4.1)	21.9 (2.3)	4.623	<.001	***
**ABC, rs**	9.78 (4.17)	13.04 (3.61)	13.141	<.001	***
**Austria weight classification**					
Underweight	46 (7.6)	21 (5.3)	p-lvl = ***<br>(see Table. S3)		
Normal weight	420 (69.0)	336 (84.8)			
Overweight	100 (16.4)	39 (9.8)			
Obesity	34 (5.6)	0 (0.0)			
Morbid obesity	9 (1.5)	0 (0.0)			

N = study population. Data are No. (%) or mean (SD). No. = number; % = percent; SD = standard deviation; kg = kilogram; cm = centimeter; BMI = body mass index; kg/m2 = kilogram/meter squared; EQUI BMI$_{AUT}$ = equivalent BMI based on Austrian reference centile curves passing through adult BMI values [1]; ABC = Austrian Balance Check; T = measurement time point; rs = raw score; *t* = *t*-test; p-lvl (P value level): * = P < 0.05, ** = P < 0.01 and *** = P < 0.001.

Table S4. Mann–Whitney U-test used to assess differences in classification groups between regular school classes and elite sports classes

Variable	Regular school class vs. elite sports school class					
	U	Z	P value	p-lvl	r	E.s.
**AUT weight classification**	106623	4.107	<.001	***	0.13	W
**STA 9 classification ABC**	68183	11.769	<.001	***	0.37	M

U = test statistic; Z = z-statistic; p-lvl (P Value level): * = P < 0.05, ** = P < 0.01 and *** = P < 0.001. r = Pearson correlation coefficient; E.s. = effect size; W = weak correlation; M = medium correlation; AUT = Austrian; STA9 = Stanine values, short for "standard nine", are a method of scaling test results on a nine-point standard scale. The Z-scores of the total achievable points were converted to a nine-point scale (STA9) with a mean of 5 and a standard deviation of 2. ABC = Austrian Balance Check.

Table S5. Gender- and age-specific mean values of raw values of Austrian Balance Check.

Age group	Gender	Mean values raw scores - Austrian Balance Check		t	*P* value	p-lvl
		Mean	SD			
7 and below	♂ (N=43)	4.91	3.16	-2.139	.036	*
	♀ (N=31)	6.68	3.95			
8 to 9	♂ (N=46)	6.85	4.28	-1.046	.30	
	♀ (N=42)	7.79	4.11			
10 to 11	♂ (N=32)	8.72	4.54	-1.153	.20	
	♀ (N=51)	9.75	2.76			
12 to 13	♂ (N=53)	10.17	3.78	-0.996	.32	
	♀ (N=39)	10.92	3.30			
14 to 15	♂ (N=43)	10.70	3.63	-0.674	.50	
	♀ (N=54)	11.17	3.21			
16 to 17	♂ (N=49)	11.57	3.09	-0.493	.62	
	♀ (N=75)	11.88	3.59			
18 to 19	♂ (N=20)	12.05	2.98	-0.669	.51	
	♀ (N=31)	12.68	3.44			

N = study population. Data are No. or mean (SD). No. = number; SD = standard deviation; *t* = *t*-test; p-lvl (P value level): * = P < 0.05, ** = P < 0.01 and *** = P < 0.001.

Table S6. Traditional z-scores and STA nine values.

Variable	Gender	Age group	Total number of points achieved (0 to 20) when performing ABC																				
			**0**	**1**	**2**	**3**	**4**	**5**	**6**	**7**	**8**	**9**	**10**	**11**	**12**	**13**	**14**	**15**	**16**	**17**	**18**	**19**	**20**
**z-score**	**Boys**	7 and below	-1.55	-1.24	-0.92	-0.60	-0.29	0.03	0.35	0.66	0.98	1.29	1.61	1.93	2.24	2.56	2.88	3.19	3.51	3.83	4.14	4.46	4.77
		8 to 9	-1.60	-1.37	-1.13	-0.90	-0.67	-0.43	-0.20	0.04	0.27	0.50	0.74	0.97	1.20	1.44	1.67	1.90	2.14	2.37	2.60	2.84	3.07
		10 to 11	-1.92	-1.70	-1.48	-1.26	-1.04	-0.82	-0.60	-0.38	-0.16	0.06	0.28	0.50	0.72	0.94	1.16	1.38	1.60	1.82	2.04	2.27	2.49
		12 to 13	-2.69	-2.43	-2.16	-1.90	-1.63	-1.37	-1.10	-0.84	-0.57	-0.31	-0.05	0.22	0.48	0.75	1.01	1.28	1.54	1.81	2.07	2.34	2.60
		14 to 15	-2.95	-2.67	-2.40	-2.12	-1.85	-1.57	-1.30	-1.02	-0.74	-0.47	-0.19	0.08	0.36	0.63	0.91	1.18	1.46	1.74	2.01	2.29	2.56
		16 to 17	-3.75	-3.42	-3.10	-2.77	-2.45	-2.13	-1.80	-1.48	-1.16	-0.83	-0.51	-0.18	0.14	0.46	0.79	1.11	1.43	1.76	2.08	2.40	2.73
		18 to 19	-4.04	-3.71	-3.37	-3.03	-2.70	-2.36	-2.03	-1.69	-1.36	-1.02	-0.69	-0.35	-0.02	0.32	0.65	0.99	1.32	1.66	2.00	2.33	2.67
	**Girls**	7 and below	-1.69	-1.44	-1.18	-0.93	-0.68	-0.42	-0.17	0.08	0.33	0.59	0.84	1.09	1.35	1.60	1.85	2.11	2.36	2.61	2.86	3.12	3.37
		8 to 9	-1.90	-1.65	-1.41	-1.17	-0.92	-0.68	-0.43	-0.19	0.05	0.30	0.54	0.78	1.03	1.27	1.51	1.76	2.00	2.24	2.49	2.73	2.98
		10 to 11	-3.54	-3.17	-2.81	-2.45	-2.08	-1.72	-1.36	-1.00	-0.63	-0.27	0.09	0.46	0.82	1.18	1.54	1.91	2.27	2.63	3.00	3.36	3.72
		12 to 13	-3.31	-3.00	-2.70	-2.40	-2.10	-1.79	-1.49	-1.19	-0.88	-0.58	-0.28	0.02	0.33	0.63	0.93	1.23	1.54	1.84	2.14	2.44	2.75
		14 to 15	-3.47	-3.16	-2.85	-2.54	-2.23	-1.92	-1.61	-1.30	-0.99	-0.67	-0.36	-0.05	0.26	0.57	0.88	1.19	1.50	1.81	2.13	2.44	2.75
		16 to 17	-3.31	-3.03	-2.75	-2.47	-2.19	-1.91	-1.64	-1.36	-1.08	-0.80	-0.52	-0.24	0.03	0.31	0.59	0.87	1.15	1.42	1.70	1.98	2.26
		18 to 19	-3.69	-3.40	-3.10	-2.81	-2.52	-2.23	-1.94	-1.65	-1.36	-1.07	-0.78	-0.49	-0.20	0.09	0.38	0.68	0.97	1.26	1.55	1.84	2.13
**STA9 values**	**Boys**	7 and below	1.90	2.53	3.16	3.79	4.43	5.06	5.69	6.32	6.96	7.59	8.22	8.86	9.49	10.12	10.75	11.39	12.02	12.65	13.28	13.92	14.55
		8 to 9	1.80	2.27	2.74	3.20	3.67	4.14	4.60	5.07	5.54	6.00	6.47	6.94	7.40	7.87	8.34	8.80	9.27	9.74	10.21	10.67	11.14
		10 to 11	1.16	1.60	2.04	2.48	2.92	3.36	3.80	4.24	4.68	5.12	5.56	6.00	6.45	6.89	7.33	7.77	8.21	8.65	9.09	9.53	9.97
		12 to 13	-0.39	0.14	0.67	1.20	1.73	2.26	2.79	3.32	3.85	4.38	4.91	5.44	5.97	6.50	7.03	7.56	8.09	8.62	9.15	9.68	10.21
		14 to 15	-0.90	-0.35	0.21	0.76	1.31	1.86	2.41	2.96	3.51	4.06	4.61	5.17	5.72	6.27	6.82	7.37	7.92	8.47	9.02	9.57	10.13
		16 to 17	-2.49	-1.84	-1.20	-0.55	0.10	0.75	1.39	2.04	2.69	3.34	3.98	4.63	5.28	5.92	6.57	7.22	7.87	8.51	9.16	9.81	10.46
		18 to 19	-3.08	-2.41	-1.74	-1.07	-0.40	0.27	0.94	1.61	2.28	2.95	3.63	4.30	4.97	5.64	6.31	6.98	7.65	8.32	8.99	9.66	10.33
	**Girls**	7 and below	1.62	2.13	2.63	3.14	3.65	4.15	4.66	5.16	5.67	6.18	6.68	7.19	7.69	8.20	8.70	9.21	9.72	10.22	10.73	11.23	11.74
		8 to 9	1.21	1.69	2.18	2.67	3.16	3.64	4.13	4.62	5.10	5.59	6.08	6.57	7.05	7.54	8.03	8.51	9.00	9.49	9.98	10.46	10.95
		10 to 11	-2.07	-1.35	-0.62	0.10	0.83	1.56	2.28	3.01	3.73	4.46	5.19	5.91	6.64	7.36	8.09	8.81	9.54	10.27	10.99	11.72	12.44
		12 to 13	-1.61	-1.01	-0.40	0.20	0.81	1.41	2.02	2.63	3.23	3.84	4.44	5.05	5.65	6.26	6.86	7.47	8.07	8.68	9.28	9.89	10.49
		14 to 15	-1.95	-1.33	-0.70	-0.08	0.54	1.16	1.78	2.41	3.03	3.65	4.27	4.90	5.52	6.14	6.76	7.39	8.01	8.63	9.25	9.87	10.50
		16 to 17	-1.61	-1.05	-0.50	0.06	0.62	1.17	1.73	2.28	2.84	3.40	3.95	4.51	5.07	5.62	6.18	6.74	7.29	7.85	8.41	8.96	9.52
		18 to 19	-2.37	-1.79	-1.21	-0.63	-0.05	0.53	1.12	1.70	2.28	2.86	3.44	4.02	4.61	5.19	5.77	6.35	6.93	7.51	8.10	8.68	9.26

z-score = traditional z standardization; STA9 = Stanine values, short for "standard nine", are a method of scaling test results on a nine-point standard scale. The Z-scores of the total achievable points were converted to a nine-point scale (STA9) with a mean of 5 and a standard deviation of 2. ABC = Austrian Balance Check

Table S7. Descriptive statistics of the results for checking objectivity.

Age group	Group	Regular school class					Elite sports school class				
		N	ABC R1 T1, rs		ABC R2 T1, rs		N	ABC R1 T1, rs		ABC R2 T1, rs	
			M	SD	M	SD		M	SD	M	SD
**All participants**	**All**	**210**	8.56	4.61	8.54	4.62	**73**	11.78	3.46	11.71	3.47
	**Boys**	**110**	7.96	4.79	7.96	4.81	**52**	11.75	3.58	11.69	3.59
	**Girls**	**100**	9.22	4.33	9.17	4.34	**21**	11.86	3.23	11.76	3.25
**7 and below**	**All**	**50**	5.78	3.86	5.42	3.90	**ND**	ND	ND	ND	ND
	**Boys**	**31**	4.84	3.41	4.42	3.37	**ND**	ND	ND	ND	ND
	**Girls**	**19**	7.32	4.14	7.05	4.22	**ND**	ND	ND	ND	ND
**8 to 9**	**All**	**35**	5.26	3.80	5.49	3.59	**ND**	ND	ND	ND	ND
	**Boys**	**19**	4.89	3.91	5.26	3.63	**ND**	ND	ND	ND	ND
	**Girls**	**16**	5.69	3.74	5.75	3.64	**ND**	ND	ND	ND	ND
**10 to 11**	**All**	**ND**	ND	ND	ND	ND	**ND**	ND	ND	ND	ND
	**Boys**	**ND**	ND	ND	ND	ND	**ND**	ND	ND	ND	ND
	**Girls**	**ND**	ND	ND	ND	ND	**ND**	ND	ND	ND	ND
**12 to 13**	**All**	**59**	10.44	3.73	10.42	3.66	**51**	11.41	3.34	11.41	3.35
	**Boys**	**32**	10.31	3.71	10.38	3.66	**37**	11.24	3.34	11.27	3.36
	**Girls**	**27**	10.59	3.83	10.48	3.72	**14**	11.86	3.42	11.79	3.42
**14 to 15**	**All**	**26**	9.08	3.65	9.19	3.64	**22**	12.64	3.66	12.41	3.72
	**Boys**	**14**	8.86	4.26	9.07	4.25	**15**	13.00	3.95	12.73	4.03
	**Girls**	**12**	9.33	2.96	9.33	2.96	**7**	11.86	3.08	11.71	3.15
**16 to 17**	**All**	**24**	10.88	4.34	10.79	4.39	**ND**	ND	ND	ND	ND
	**Boys**	**7**	11.14	4.34	11.00	4.32	**ND**	ND	ND	ND	ND
	**Girls**	**17**	10.76	4.47	10.71	4.55	**ND**	ND	ND	ND	ND
**18 to 19**	**All**	**16**	13.25	3.45	13.56	3.31	**ND**	ND	ND	ND	ND
	**Boys**	**7**	14.43	3.36	14.71	3.30	**ND**	ND	ND	ND	ND
	**Girls**	**9**	12.33	3.43	12.67	3.20	**ND**	ND	ND	ND	ND

N = study population. Data are mean (SD). No. = number; SD = standard deviation; ABC = Austrian Balance Check; T = measurement time point; rs = raw score; T1 = baseline measurements made in autumn 2023; T2 = measurement taken within 2 weeks of baseline measurement; R1 = Rater 1; R2 = Rater 2; ND = no data.

.

Table S8. Descriptive statistics of the results for checking reliability using the test–retest method.

Age group	Group	Regular school class					Elite sports school class				
		N	ABC T1, rs		ABC T2, rs		N	ABC T1, rs		ABC T2, rs	
			M	SD	M	SD		M	SD	M	SD
**All participants**	**All**	**342**	9.74	4.39	9.77	4.48	**139**	12.37	3.50	12.79	3.43
	**Boys**	**161**	9.07	4.70	8.88	4.57	**104**	12.25	3.42	12.70	3.36
	**Girls**	**181**	10.33	4.03	10.57	4.26	**35**	12.74	3.75	13.06	3.65
**7 and below**	**All**	**48**	5.81	3.98	5.81	4.00	**ND**	ND	ND	ND	ND
	**Boys**	**29**	4.86	3.50	5.10	3.55	**ND**	ND	ND	ND	ND
	**Girls**	**19**	7.26	4.31	6.58	4.71	**ND**	ND	ND	ND	ND
**8 to 9**	**All**	**35**	5.86	3.83	5.37	3.99	**ND**	ND	ND	ND	ND
	**Boys**	**17**	5.06	4.10	4.76	4.31	**ND**	ND	ND	ND	ND
	**Girls**	**18**	6.61	3.50	6.33	3.25	**ND**	ND	ND	ND	ND
**10 to 11**	**All**	**35**	9.77	3.40	10.09	3.31	**35**	12.34	3.30	12.94	3.19
	**Boys**	**14**	8.79	3.85	8.93	3.58	**26**	12.00	3.02	12.58	3.04
	**Girls**	**21**	10.43	2.99	10.86	2.95	**9**	13.33	4.03	14.00	3.57
**12 to 13**	**All**	**60**	10.45	3.70	10.55	3.69	**62**	11.76	3.51	12.18	3.40
	**Boys**	**32**	10.31	3.71	10.22	3.80	**47**	11.55	3.39	12.09	3.28
	**Girls**	**28**	10.61	3.76	10.93	3.60	**15**	12.40	3.91	12.47	3.87
**14 to 15**	**All**	**55**	10.53	3.49	10.36	3.67	**25**	12.60	3.59	12.96	3.17
	**Boys**	**25**	10.04	3.88	9.56	3.69	**18**	12.89	3.82	13.28	3.32
	**Girls**	**30**	10.93	3.14	11.03	3.59	**7**	11.86	3.08	12.14	2.80
**16 to 17**	**All**	**73**	11.64	4.12	11.75	4.01	**16**	14.38	3.32	14.44	4.11
	**Boys**	**26**	11.85	4.40	11.27	3.86	**12**	14.42	3.12	14.58	3.99
	**Girls**	**47**	11.53	4.01	12.02	4.10	**4**	14.25	4.43	14.00	4.32
**18 to 19**	**All**	**36**	12.47	3.34	12.83	3.28	**ND**	ND	ND	ND	ND
	**Boys**	**18**	12.33	3.45	12.33	3.11	**ND**	ND	ND	ND	ND
	**Girls**	**18**	12.61	3.33	13.33	3.46	**ND**	ND	ND	ND	ND

N = study population. Data are mean (SD). No. = number; SD = standard deviation; ABC = Austrian Balance Check; T = measurement time point; rs = raw score; ND = no data.

Table S9. Reliability and objectivity of ABC performance in different age groups and separately for boys and girls

Test quality criteria	Age group	Group	Regular school class					Elite sports school class				
			N	ICC (2.1)	95% CI		Agreement	N	ICC (2.1)	95% CI		Agreement
					Lower	Upper				Lower	Upper	
Test of Reliability	7 and below	**All**	**48**	0.895	0.820	0.940	Good to Excellent	**ND**	ND	ND	ND	-
		**Boys**	**29**	0.904	0.808	0.954	Good to Excellent	**ND**	ND	ND	ND	-
		**Girls**	**19**	0.878	0.715	0.951	Moderate to Excellent	**ND**	ND	ND	ND	-
	8 to 9	**All**	**35**	0.856	0.734	0.924	Moderate to Excellent	**ND**	ND	ND	ND	-
		**Boys**	**17**	0.824	0.588	0.932	Moderate to Excellent	**ND**	ND	ND	ND	-
		**Girls**	**18**	0.895	0.746	0.959	Moderate to Excellent	**ND**	ND	ND	ND	-
	10 to 11	**All**	**35**	0.842	0.711	0.917	Moderate to Excellent	**35**	0.918	0.812	0.962	Good to Excellent
		**Boys**	**14**	0.843	0.578	0.947	Moderate to Excellent	**26**	0.907	0.779	0.960	Good to Excellent
		**Girls**	**21**	0.825	0.624	0.925	Moderate to Excellent	**9**	0.939	0.753	0.986	Good to Excellent
	12 to 13	**All**	**60**	0.946	0.912	0.968	Excellent	**62**	0.862	0.779	0.915	Good to Excellent
		**Boys**	**32**	0.932	0.866	0.966	Good to Excellent	**47**	0.866	0.766	0.925	Good to Excellent
		**Girls**	**28**	0.964	0.923	0.984	Excellent	**15**	0.855	0.620	0.949	Moderate to Excellent
	14 to 15	**All**	**55**	0.838	0.737	0.902	Moderate to Excellent	**25**	0.932	0.853	0.970	Good to Excellent
		**Boys**	**25**	0.807	0.614	0.910	Moderate to Excellent	**18**	0.933	0.833	0.974	Good to Excellent
		**Girls**	**30**	0.866	0.737	0.934	Moderate to Excellent	**7**	0.934	0.689	0.988	Moderate to Excellent
	16 to 17	**All**	**73**	0.905	0.852	0.939	Good to Excellent	**16**	0.894	0.723	0.962	Moderate to Excellent
		**Boys**	**26**	0.903	0.794	0.955	Good to Excellent	**12**	0.86	0.580	0.958	Moderate to Excellent
		**Girls**	**47**	0.908	0.838	0.948	Good to Excellent	**4**	0.988	0.831	0.999	Good to Excellent
	18 to 19	**All**	**36**	0.865	0.752	0.929	Good to Excellent	**ND**	ND	ND	ND	-
		**Boys**	**18**	0.933	0.829	0.974	Good to Excellent	**ND**	ND	ND	ND	-
		**Girls**	**18**	0.807	0.562	0.923	Moderate to Excellent	**ND**	ND	ND	ND	-
Test of Objectivity	7 and below	**All**	**50**	0.948	0.908	0.971	Excellent	**ND**	ND	ND	ND	-
		**Boys**	**31**	0.901	0.804	0.951	Good to Excellent	**ND**	ND	ND	ND	-
		**Girls**	**19**	0.989	0.970	0.996	Excellent	**ND**	ND	ND	ND	-
	8 to 9	**All**	**35**	0.96	0.923	0.980	Excellent	**ND**	ND	ND	ND	-
		**Boys**	**19**	0.958	0.894	0.983	Good to Excellent	**ND**	ND	ND	ND	-
		**Girls**	**16**	0.965	0.904	0.988	Excellent	**ND**	ND	ND	ND	-
	10 to 11	**All**	**ND**	ND	ND	ND	-	**ND**	ND	ND	ND	-
		**Boys**	**ND**	ND	ND	ND	-	**ND**	ND	ND	ND	-
		**Girls**	**ND**	ND	ND	ND	-	**ND**	ND	ND	ND	-
	12 to 13	**All**	**59**	0.993	0.989	0.996	Excellent	**51**	0.981	0.966	0.989	Excellent
		**Boys**	**32**	0.993	0.986	0.997	Excellent	**37**	0.975	0.951	0.987	Excellent
		**Girls**	**27**	0.994	0.986	0.997	Excellent	**14**	0.997	0.991	0.999	Excellent

	14 to 15	**All**	**26**	0.996	0.990	0.998	Excellent	**22**	0.965	0.919	0.985	Excellent
		**Boys**	**14**	0.994	0.980	0.998	Excellent	**15**	0.958	0.883	0.986	Good to Excellent
		**Girls**	**12**	>.99	>.99	>.99	Excellent	**7**	0.993	0.963	0.999	Excellent
	16 to 17	**All**	**24**	0.998	0.995	0.999	Excellent	**ND**	ND	ND	ND	-
		**Boys**	**7**	0.996	0.981	0.999	Excellent	**ND**	ND	ND	ND	-
		**Girls**	**17**	0.999	0.996	0.999	Excellent	**ND**	ND	ND	ND	-
	18 to 19	**All**	**16**	0.986	0.945	0.996	Excellent	**ND**	ND	ND	ND	-
		**Boys**	**7**	0.987	0.928	0.998	Excellent	**ND**	ND	ND	ND	-
		**Girls**	**9**	0.985	0.919	0.997	Excellent	**ND**	ND	ND	ND	-

Explanation: To determine the reliability of the test [ABC T1]–retest [ABC T2]) method and the objectivity (test rater 1 [ABC R1 T1] – test rater 2 [ABC R2 T2]); the 95% CIs of the ICCs were interpreted as follows: 95% CI values below 0.5 were considered to indicate poor reliability, values between 0.5 and 0.75 were considered to indicate moderate reliability, values between 0.75 and 0.9 were considered to indicate good reliability, and values above 0.90 were considered to indicate excellent reliability. ICC = intraclass correlation; CI = confidence interval; ABC = Austrian Balance Check; ND = no data.

Table S10. Descriptive statistics of the results for checking validity using the KINVENT force plate.

Age group	Group	Regular school class				
		N	ABC T3. rs	FP SLS. rs		
				COP Su, mm2	COP Mv. mm/s	COP Pl. mm
**All participants**	**All**	**163**	12.08 (3.52)	3463 (1451)	152.9 (44.7)	776 (228)
	**Boys**	**67**	10.66 (2.90)	4025 (1726)	176.8 (50.7)	899 (258)
	**Girls**	**96**	13.07 (3.58)	3071 (1069)	136.2 (30.7)	690 (156)
**7 and below**	**All**	**6**	11.50 (4.37)	2657 (788)	161.7 (34.4)	819 (175)
**8 to 9**	**All**	**7**	10.14 (2.91)	4190 (2089)	156.6 (39.5)	792 (198)
**10 to 11**	**All**	**24**	11.00 (3.01)	3532 (1508)	159.9 (36.9)	812 (188)
**12 to 13**	**All**	**31**	11.16 (3.03)	3584 (1417)	158.8 (46.8)	805 (238)
**14 to 15**	**All**	**33**	12.15 (3.43)	3575 (1271)	155.6 (47.1)	794 (244)
**16 to 17**	**All**	**46**	13.70 (3.75)	3217 (1576)	143.5 (48.9)	727 (247)
**18 to 19**	**All**	**16**	11.75 (3.26)	3581 (1274)	147.6 (41.3)	748 (209)

N = study population. Data are mean (SD). SD = standard deviation; ABC = Austrian Balance Check; T = measurement time point; rs = raw score; FB = force plate; SLS = single-leg stand; COP = center of pressure; Su = surface; Mv = medium velocity; Pl = path length; mm^2 = square millimeter; mm/s = millimeters per second.

Table S11. Age-specific data from the validity check using Spearman's correlation coefficient.

Age group	Variable		Regular school class			
				FP SLS. rs		
			ABC T3. rs	COP Su, mm2	COP Mv. mm/s	COP Pl. mm
7 and below	ABC T3. rs		--			
	FP SLS. rs	Surface. mm2	-0.883*	--		
		COP Mv. mm/s	-0.794	0.886*	--	
		COP Pl. mm	-0.794	0.886*	1.000**	--
8 to 9	ABC T3. rs		--			
	FP SLS. rs	Surface. mm2	-0.786*	--		
		COP Mv. mm/s	-0.929**	0.607	--	
		COP Pl. mm	-0.929**	0.607	1.000**	--
10 to 11	ABC T3. rs		--			
	FP SLS. rs	Surface. mm2	-0.536**	--		
		COP Mv. mm/s	-0.604**	0.545**	--	
		COP Pl. mm	-0.598**	0.541**	0.998**	--
12 to 13	ABC T3. rs		--			
	FP SLS. rs	Surface. mm2	-0.568**	--		
		COP Mv. mm/s	-0.624**	0.640**	--	
		COP Pl. mm	-0.623**	0.638**	1.000**	--
14 to 15	ABC T3. rs		--			
	FP SLS. rs	Surface. mm2	-0.686**	--		
		COP Mv. mm/s	-0.691**	0.740**	--	
		COP Pl. mm	-0.688**	0.736**	0.998**	--
16 to 17	ABC T3. rs		--			
	FP SLS. rs	Surface. mm2	-.519**	--		
		COP Mv. mm/s	-.618**	0.752**	--	
		COP Pl. mm	-.607**	.748**	.998**	--
18 to 19	ABC T3. rs		--			
	FP SLS. rs	Surface. mm2	-.649**	--		
		COP Mv. mm/s	-.516*	.809**	--	
		COP Pl. mm	-.514*	.815**	.999**	--

* = correlation is significant at the 0.05 level (2-tailed); ** = correlation is significant at the 0.01 level (2-tailed); ABC = Austrian Balance Check; T3 = measuring timing of ABC on same day as measuring of balance using electronic force plate; rs = raw score; FB = force plate; SLS = single-leg stand; COP = center of pressure; Su = surface; Mv = medium velocity; Pl = path length; mm^2 = square millimeter; mm/s = millimeters per second.

Table S12. Age- and gender-specific data from the competitiveness review using Spearman's correlation coefficient.

Age group	Group	Regular school class							Elite sports school class						
		N	ABC R1 T1, rs		BB GMT, rs		ρ	E.s.	N	ABC R1 T1, rs		BB GMT, rs		ρ	E.s.
			M	SD	M	SD				M	SD	M	SD		
**All participants**	**All**	**458**	9.41	4.20	29.25	11.50	,519**	S	**279**	13.01	3.64	40.71	7.43	,338**	M
	**Boys**	**223**	8.85	4.47	26.98	12.26	,538**	S	**214**	12.88	3.64	39.78	7.63	,308**	M
	**Girls**	**235**	9.94	3.85	31.41	10.31	,454**	M	**65**	13.43	3.63	43.77	5.81	,394**	M
**7 and below**	**All**	**69**	5.70	3.62	25.48	10.22	,486**	M	**ND**	ND	ND	ND	ND	ND	ND
	**Boys**	**39**	4.79	3.18	21.62	8.87	0.269	M	**ND**	ND	ND	ND	ND	ND	ND
	**Girls**	**30**	6.87	3.88	30.50	9.78	,595**	S	**ND**	ND	ND	ND	ND	ND	ND
**8 to 9**	**All**	**79**	7.37	4.14	29.10	11.47	,552**	S	**29**	10.66	3.75	38.59	6.44	,392*	M
	**Boys**	**39**	6.74	4.23	28.67	12.97	,674**	S	**23**	10.52	3.95	37.30	6.06	0.343	M
	**Girls**	**40**	7.98	4.02	29.53	9.93	,423**	M	**6**	11.17	3.13	43.50	5.86	0.616	S
**10 to 11**	**All**	**71**	9.39	3.34	29.30	11.45	,541**	S	**63**	11.17	3.39	40.17	7.76	,351**	M
	**Boys**	**26**	8.77	4.34	26.46	12.22	,619**	S	**46**	10.83	3.13	39.37	8.32	,384**	M
	**Girls**	**45**	9.76	2.58	30.93	10.79	,440**	M	**17**	12.12	3.97	42.35	5.62	0.215	W
**12 to 13**	**All**	**87**	10.36	3.55	25.59	12.04	,375**	M	**46**	13.48	3.42	38.61	8.53	0.281	W
	**Boys**	**51**	10.04	3.73	23.41	12.93	,352*	M	**34**	13.47	3.34	37.82	8.73	0.287	W
	**Girls**	**36**	10.81	3.29	28.67	10.04	0.302	M	**12**	13.50	3.78	40.83	7.85	0.315	M
**14 to 15**	**All**	**60**	11.13	3.34	30.88	11.11	,636**	S	**105**	14.07	3.21	41.95	7.06	,257**	W
	**Boys**	**26**	10.54	3.59	29.65	12.77	,878**	S	**85**	14.00	3.21	41.06	7.19	0.185	W
	**Girls**	**34**	11.59	3.12	31.82	9.75	,395*	M	**20**	14.35	3.25	45.75	5.11	,526*	S
**16 to 17**	**All**	**69**	12.06	3.77	34.09	10.59	,501**	S	**36**	14.42	3.43	42.39	6.29	,582**	S
	**Boys**	**32**	11.88	3.37	31.56	10.24	,471**	M	**26**	14.15	3.62	41.04	6.82	,654**	S
	**Girls**	**37**	12.22	4.13	36.27	10.53	,510**	S	**10**	15.10	2.96	45.90	2.38	0.204	W
**18 to 19**	**All**	**23**	11.61	3.01	36.09	8.38	,418*	M	**ND**	ND	ND	ND	ND	ND	ND
	**Boys**	**10**	13.00	3.59	39.30	5.60	0.459	M	**ND**	ND	ND	ND	ND	ND	ND
	**Girls**	**13**	10.54	2.03	33.62	9.48	0.273	W	**ND**	ND	ND	ND	ND	ND	ND

N = study population. Data are No. (%) or mean (SD). No. = number; % = percent; SD = standard deviation; ABC = Austrian Balance Check; BB = balancing backwards; GMT = German Motor Test; T = measurement time point; R = rater; rs = raw score. Spearman's correlation coefficient (ρ) = $\rho \geq 0.10$ to 0.29 = weak correlation; $\rho \geq 0.30$ to 0.49 moderate correlation; $\rho > 0.50$ strong correlation. Level of significance = * = $P < 0.05$, ** = $P < 0.01$ and *** = $P < 0.001$. W = weak correlation; M = medium correlation; S = strong correlation; ND = no data.

Table S13. Comparison of testing times between ABC and BB GMT by using paired t-test.

	**Pairwise comparisons**	**Paired Differences**		**t**	**df**	**P value**	**E.s.**
		**Mean diff. (95% CI)**	**S.E.**				
**Regular school class**	**Time ABC - T1 - BB GMT**	-1.27 (-1.29 to -1.26)	0.01	-141.978	457	<.001	L
**Elite sports school class**	**Time ABC - T1 - BB GMT**	-1.30 (-1.31 to -1.28)	0.01	-144.878	278	<.001	L

The data show the mean difference in testing times measured in minutes per participant. Mean diff = mean difference; CI = confidence interval; SE = standard error; t = test statistic; df = degree of freedom; P value means = *** $p < .001$; E.s. = effect size; L = large effect according to Cohen ($d \geq 0.8$). ABC = Austrian Balance Check; BB = balancing backwards; GMT = German Motor Test; T = measurement time point.

Figure S1. Gender- and age-specific mean values of raw values of Austrian Balance Check.

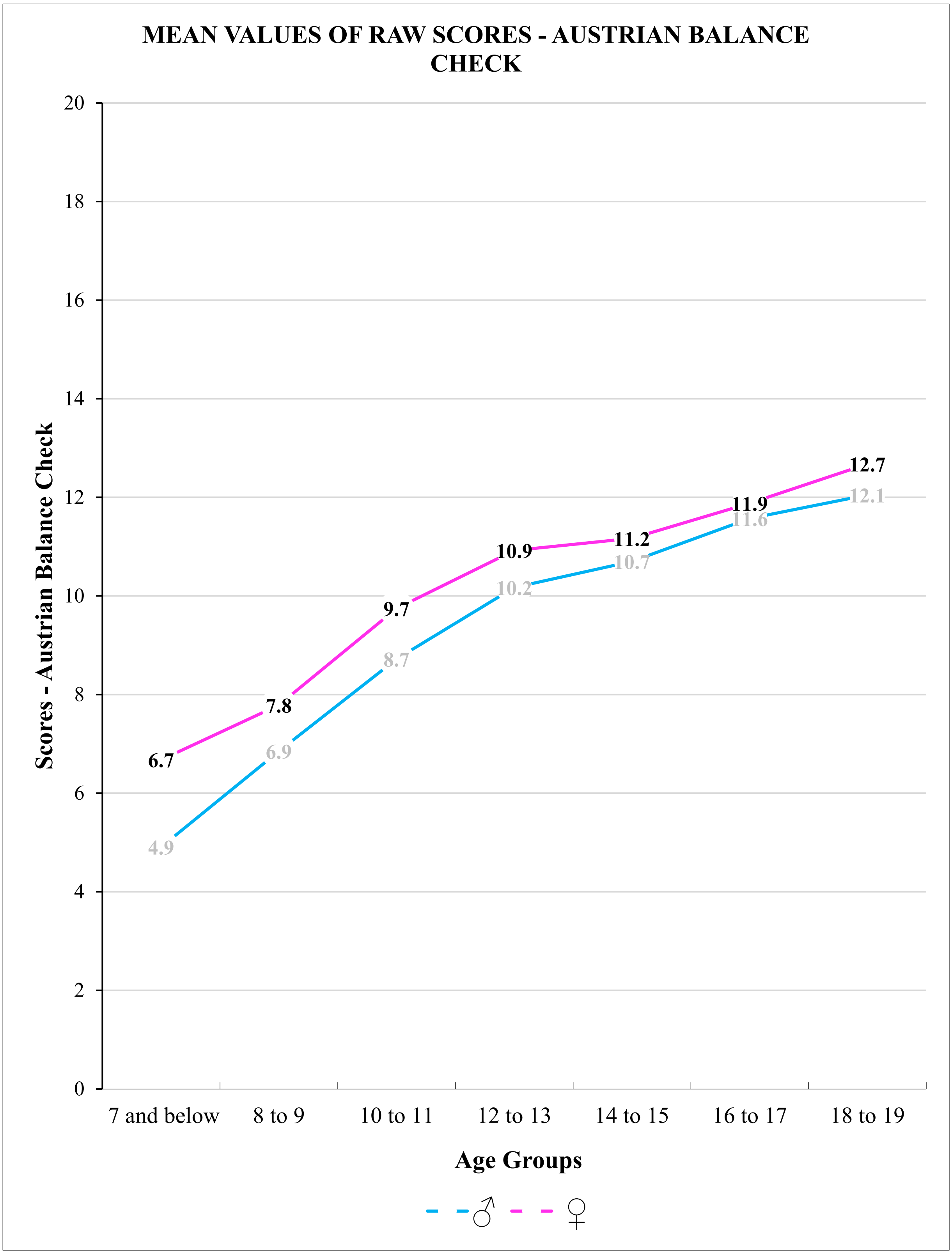

Figure S2. A graphical illustration of the normal distribution using histograms for the classification of the Austrian Balance Check and balancing backwards (German Motor Test).

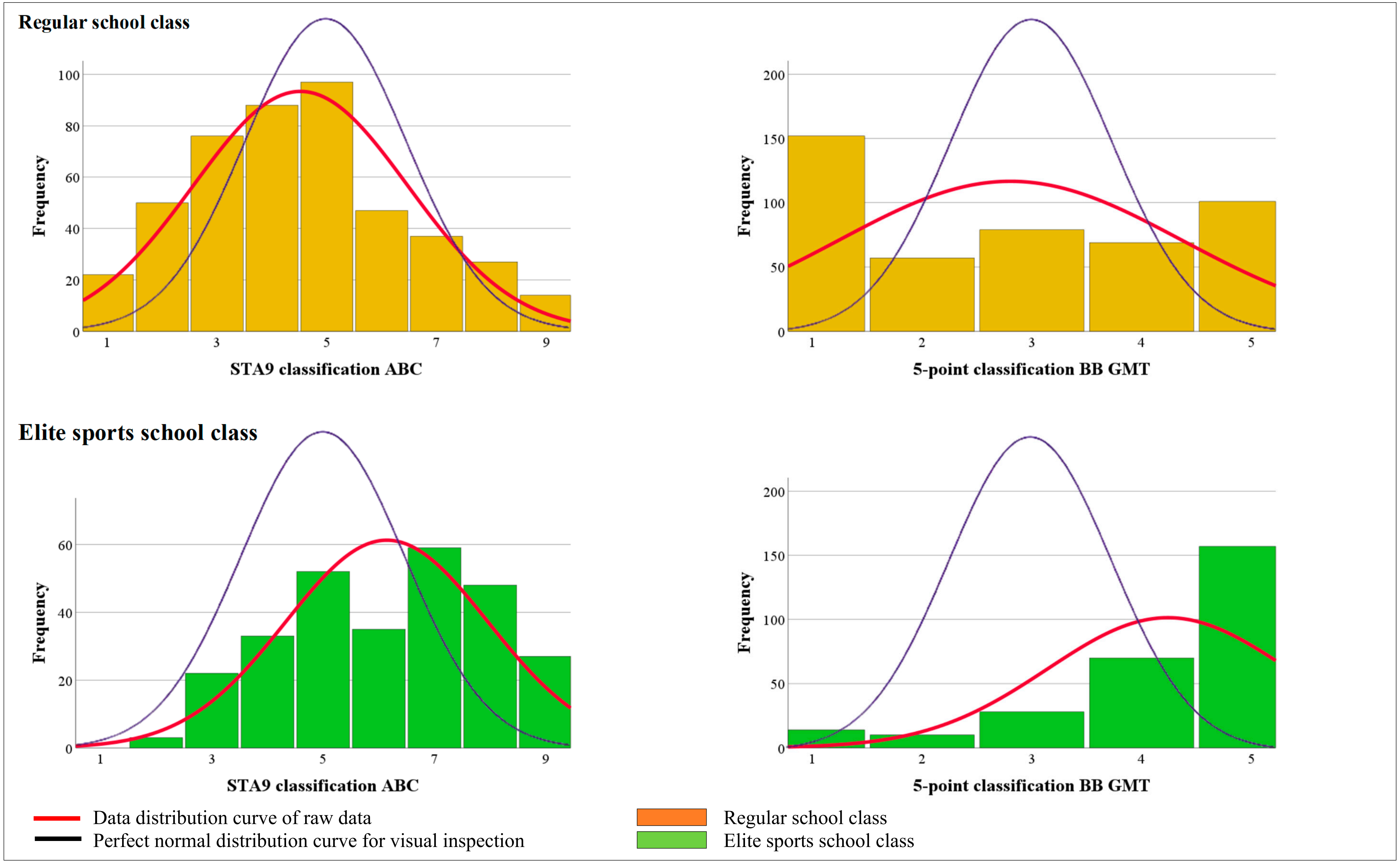

REFERENCES

1 Mayer M, Gleiss A, Häusler G, et al. Weight and body mass index (BMI): current data for Austrian boys and girls aged 4 to under 19 years. *Ann Hum Biol* 2015;42(1):45–55.
